# Synthesis of mono-nitroxides and of bis-nitroxides with varying electronic through-bond communication†

**DOI:** 10.1039/d2ob01863b

**Published:** 2022-12-08

**Authors:** Angeliki Giannoulis, Katrin Ackermann, Alexey Bogdanov, David B. Cordes, Catherine Higgins, Joshua Ward, Alexandra M. Z. Slawin, James E. Taylor, Bela E. Bode

**Affiliations:** a Department of Chemical and Biological Physics, Weizmann Institute of Science Rehovot 76100 Israel angeliki.giannoulis@weizmann.ac.il; b EaStCHEM School of Chemistry, Biomedical Sciences Research Complex and Centre of Magnetic Resonance, University of St Andrews North Haugh St Andrews KY16 9ST UK beb2@st-andrews.ac.uk; c Department of Chemistry, University of Bath Claverton Down Bath BA2 7AY UK j.e.taylor@bath.ac.uk

## Abstract

Nitroxides are a unique class of persistent radicals finding a wide range of applications, from spin probes to polarizing agents, and recently bis-nitroxides have been used as proof-of-concept molecules for quantum information processing. Here we present the syntheses of pyrroline-based nitroxide (NO) radicals and give a comparision of two possible synthetic routes to form two key intermediates, namely 2,2,5,5-tetramethylpyrroline-1-oxyl-3-acetylene (TPA) and 1-oxyl-2,2,5,5-tetramethylpyrroline-3-carboxylic acid (TPC). TPC and TPA were then used as precursors for the synthesis of three model compounds featuring two distant NO groups with a variable degree of conjugation and thus electronic communication between them. Using relatively facile synthetic routes, we produced a number of mono- and bis-nitroxides with the structures of multiple compounds unambiguously characterized by X-ray crystallography, while Continuous Wave Electron Paramagnetic Resonance (CW-EPR) allowed us to quantify the electronic communication in the bis-nitroxides. Our study expands the repertoire of mono- and bis-nitroxides with possibilities of exploiting them for studying quantum coherence effects and as polarizing agents.

## Abstract 2

The electron–electron exchange interaction (*J*) underpins quantum coherence phenomena. Here, synthetic strategies to moderate *J* in model compounds were evaluated with electron paramagnetic resonance spectroscopy.

## Graphical abstract

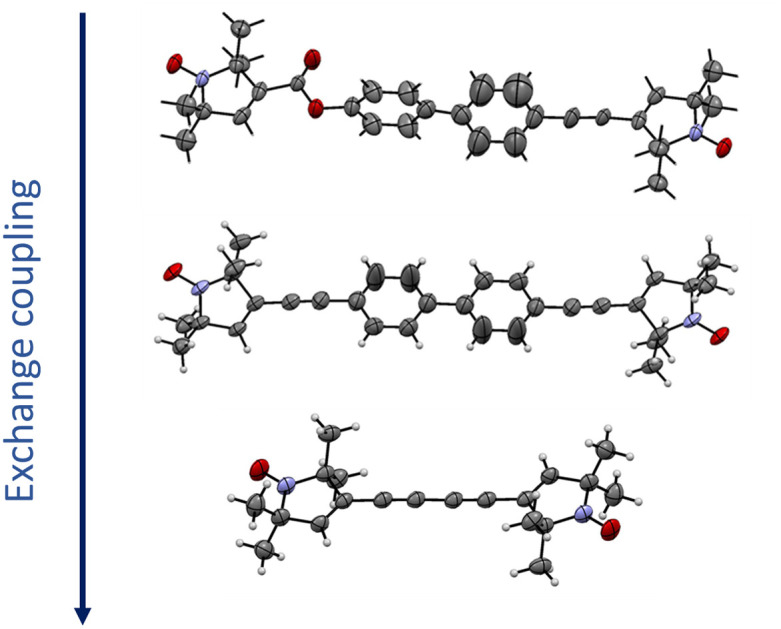

## Introduction

Electronic communication is a common effect in nature; for example, electron transfer in proteins can lead to charge separation and subsequent recombination.^1^ In general, high π-conjugation favours efficient electronic communication and possibly enables coherent quantum pathways between the electrons. Towards understanding quantum coherence (QC) phenomena, proof-of-concept paramagnetic molecules have been designed and electronic communication has been studied *via* electron paramagnetic resonance (EPR) spectroscopy which allows direct measurement of the exchange energy between two singly occupied molecular orbitals.^2,3^ In recent years pulse dipolar EPR spectroscopy (PDS) has also emerged to analyse such molecules for quantum information processing.^2,4,5^ For example, three molecular assemblies with similar macromolecular structure and flexibility differing only in the degree of the electronic communication *via* π-conjugation were used as proof-of-principle compounds for demonstrating constructive QC where a four-fold increase of the exchange coupling (*J*) was found using two conjugated linkers rather than a single one, a value in accordance with the theoretical increase in conductance.^4^ Such experiments have been sparked by new methodologies for creating and analysing different magnitudes of weak exchange interactions and their distribution.^6–8^ Additionally, the exchange interaction is of interest in dynamic nuclear polarization (DNP) experiments, where the nuclear magnetic resonance (NMR) spectroscopy sensitivity is enhanced by applying microwave irradiation to samples containing electron spins. Therefore, bis-nitroxides with a small but non-negligible *J* along with orthogonal geometry of the paramagnetic moieties determined by design are under development for DNP applications.^9^

**Fig. 1:**
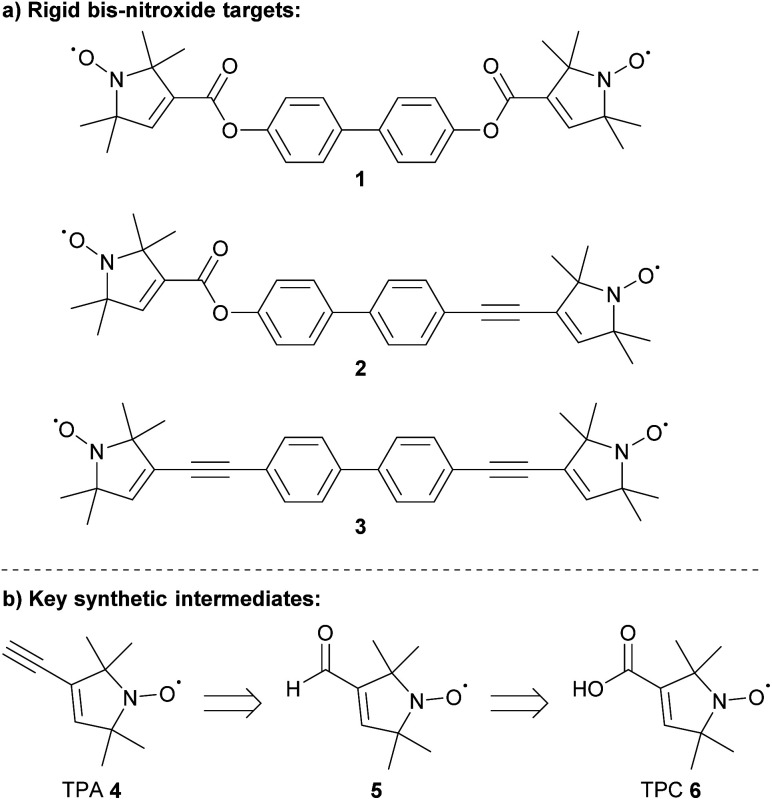
(a) Bis-nitroxide targets and (b) key intermediates.

Here we were particularly interested in the effect of ester *versus* acetylene linkages in breaking conjugation on a 2 nm scale. While double ester-linked model systems were shown to suppress the exchange coupling present in double ethyne linked analogues,^3^ the introduction of a single ester does not appear to fully diminish the exchange coupling but to considerably lessen it.^10,11^ Thus, we set out to revisit the synthesis of the ethyne- and carboxylic acid-functionalised spin-labels 2,2,5,5-tetramethyl-pyrrolin-1-oxyl-3-acetylene (TPA) and 1-oxyl-2,2,5,5-tetramethylpyrroline-3-carboxylic acid (TPC) and their use in producing three model systems with different levels of conjugation between the radical bearing pyrroline moieties. Besides the different degree of π-conjugation between the nitroxides (NOs), the bis-nitroxides feature very similar geometries and two electron spins per molecule. The rod-like geometry is afforded by the biphenyl core and the conjugation degree is tuned by attaching different combinations of ester or ethyne groups at the central biphenyl bridge (Fig. 1a). As *J* is dependent on orbital overlap it will decay exponentially with the distance between the unpaired electrons. However, exchange can be mediated through the σ- or π-bond network (or both) and this makes predictions inherently difficult and extended conjugation has been shown to remain measurable over several nm.^12^ 5-Ring nitroxides (*e.g.*, pyrrolidine-based) have been shown to retain larger conjugation and *J* than identical linkers with 6-ring-based nitroxides and were selected in this case. Predictions based on the linking groups are equally challenging as σ- or π-interactions will be attenuated differently by *e.g.*, insertion of a CH_2_ group.^13^ Computational predictions are hampered by the very small magnitude of long-range exchange couplings that lie within the numeric noise of common *ab initio* and DFT calculations. Nevertheless, the π-conjugation between biphenyl bridged pyrroline-nitroxides is expected to be significantly reduced when replacing ethyne linkages by esters. Thus, the π-conjugation and therefore *J* is expected to follow the trend: 1 < 2 < 3.

A key synthetic intermediate in the synthesis of linear bis-nitroxides 1–3 is TPA 4 ^14–18^ (Fig. 1b), which can be introduced *via* palladium-catalysed Sonogashira cross-coupling to form spin-labelled substrates suitable for EPR studies.^16,19–25^ The alkyne within TPA 4 has previously been installed through elimination of the corresponding vinyl chloride using either lithium diisopropylamide (LDA)^18^ or potassium *tert*-butoxide.^16^ Hideg and co-workers prepared TPA 4*via* 1,2-bromination of the corresponding vinyl nitroxide, followed by double elimination using potassium hydroxide in ethanol.^15^ Alternatively, TPA 4 can be prepared directly from aldehyde 5 (Fig. 1b) using the Ohira–Bestmann reagent.^17^ Nitroxide 5 can itself be obtained from either TPC 6 or its amide derivatives.^26–29^

Herein, we report the syntheses and characterisation of rigid bis-nitroxides 1–3. Two synthetic routes for the preparation of key intermediates TPA 4 and TPC 6 are investigated and compared. The key intermediates are then used in Sonogashira cross-coupling reactions and Steglich esterifications, respectively, for the efficient preparation of the bis-nitroxide targets. Most of the key intermediates and the linear bis-nitroxide products were characterised by X-ray crystallographic analysis.

## Results and discussion

### Synthesis of TPA 4 and aldehyde 5

**Scheme 1:**
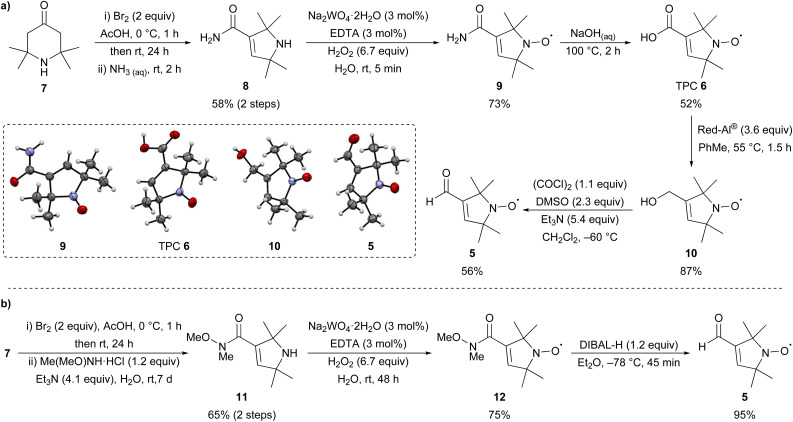
Two routes for the synthesis of nitroxide 5 (a) *via* TPC 6 and (b) *via* Weinreb amide 11.

First, nitroxide 5 was prepared from commercially available 2,2,6,6-tetramethyl-4-piperidone 7 following known literature procedures (Scheme 1a).^14,16,30,31^ Bromination followed by Favorskii rearrangement with aqueous ammonia could be performed on a large scale (77 mmol, 12 g) to give amide 8 in good yield over the two steps. Oxidation with hydrogen peroxide in the presence of catalytic sodium tungstate (3 mol%) and EDTA gave nitroxide 9 after purification by recrystallisation in 73% yield. Aqueous hydrolysis of 9 gave TPC 6, which is a synthetically useful precursor for the introduction of nitroxide spin labels *via* amidation or esterification.^32^ Reduction of TPC 6 with Red-Al®^16^ gave alcohol 10 with subsequent Swern oxidation^16^ giving the desired aldehyde 5 in good yield over the two steps. The structures of nitroxides 5, 6, a mixture of 8 and 9, and 10 were unambiguously confirmed by single crystal X-ray analysis.^33^ Although this route gave access to the desired aldehyde 5, an alternative and more succinct pathway was sought to improve the overall yield (Scheme 1b). Bromination of 7 followed by Favorskii rearrangement in the presence of *N*,*O*-dimethylhydroxylamine hydrochloride gave Weinreb amide 11 in 65% yield over the two steps,^30,34^ which was oxidised as previously to give nitroxide 12 in 75% yield. Reduction of 12 with DIBAL-H proceeded smoothly^30^ on a multi-gram scale to give aldehyde 5 (17 mmol, 2.8 g) in an excellent 95% yield. Overall, while the first route (Scheme 1a) gives access to 5*via* synthetically versatile TPC 6, aldehyde 5 is itself most conveniently prepared on a gram-scale in four steps from commercial 7 using the route depicted in Scheme 1b.

**Scheme 2:**
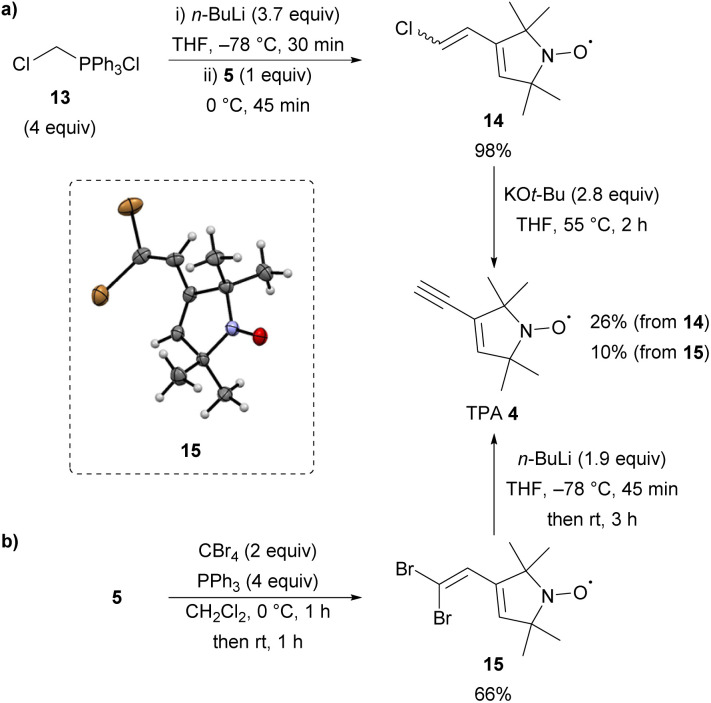
Synthesis of TPA 4 (a) *via* Literature procedure (ref. 14, 16) and (b) *via* Corey-Fuchs conditions (ref. 35).

Next, the synthesis of TPA 4 from aldehyde 5 was investigated using a literature procedure.^14,16^ Reaction of 5 with an excess of the ylide derived from chlorotriphenylphosphonium chloride 13 gave chloro-alkene 14, previously reported as a 1 : 1 mixture of *E*/*Z* stereoisomers,^18^ in an excellent 98% yield (Scheme 2a). Elimination from nitroxide 14 using KO*t*-Bu in THF gave access to TPA 4 after purification by column chromatography. Although this route gave access to the desired spin-label 4, the yield of the elimination step was variable upon multiple repeats. Moreover, intermediate nitroxide 14 was obtained as viscous yellow oil, which proved difficult to handle. Therefore, a new route to TPA 4 from aldehyde 5 was explored. Treating 5 under standard Corey–Fuchs conditions^35^ using an excess of carbon tetrabromide and triphenylphosphine gave dibromide 15 in 66% yield (Scheme 2b). Pleasingly, nitroxide 15 is a crystalline solid, which was unambiguously characterised by single-crystal X-ray analysis,^36^ and is significantly easier to handle compared with 14. Treating 15 with two equivalents of *n*-butyl lithium resulted in elimination to form TPA 4 in 10% yield. While the yield of the elimination was low, this new route proceeds *via* a more favourable intermediate and provides access to sufficient quantities of TPA 4 for further reactions.

### Synthesis of linear bis-nitroxides 1–3

**Scheme 3:**
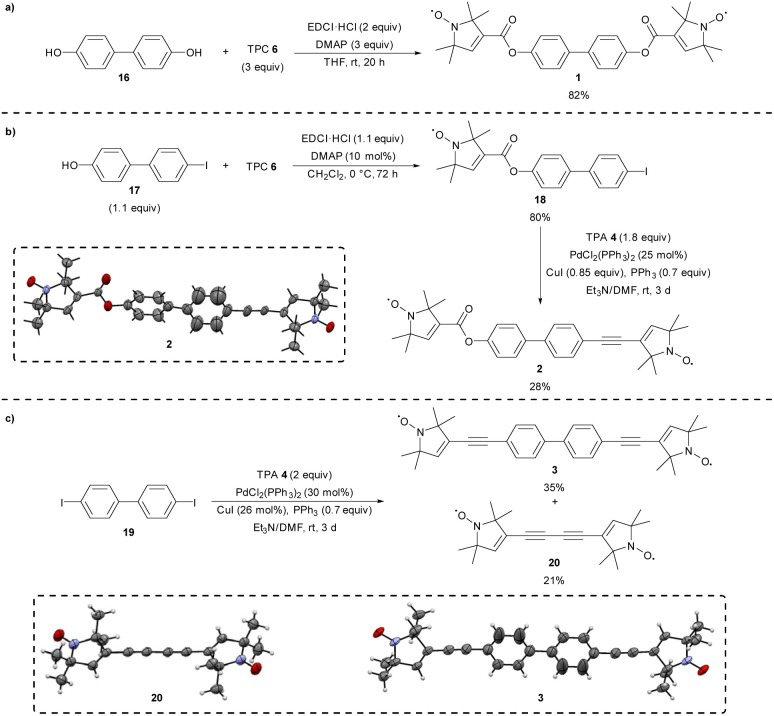
Synthesis of bis-nitroxide (a) 1, (b) 2, and (c) 3 and 20.

Having compared synthetic routes towards the synthesis of the key nitroxide spin labels 4 and 5, we turned to the preparation of linear bis-nitroxides 1–3 with differing degrees of conjugation between the two radicals. Diester 1 was prepared as described previously in 82% yield through double Steglich esterification of biphenol 16 with TPC 6 using *N*-(3-dimethylaminopropyl)-*N*′-ethylcarbodiimide hydrochloride (EDCI·HCl) as the coupling reagent (Scheme 3a).^32^ Synthesis of the linear nitroxide 2 is for the first time described here using a two-step procedure starting from 4′-iodo-[1,1′-biphenyl]-4-ol 17 (Scheme 3b). Esterification of phenol 17 with TPC 6 using EDCI·HCl proceeded smoothly, forming intermediate 18 in 80% yield.^37^ Sonogashira cross-coupling of intermediate 18 with TPA 4 gave the desired bis-nitroxide 2 in a reasonable 28% yield after purification by column chromatography, with the structure confirmed by single crystal X-ray analysis.^38^ Diethyne 3 was synthesized similarly as described previously,^3^*i.e.*, by reacting 4,4′-diiodo-1,1′-biphenyl 19 under similar Sonogashira conditions as for 2, using two equivalents of TPA 4 (Scheme 3c). Purification of 3 from the undesired homocoupled product of TPA 4 was found to be difficult, nonetheless separation was achieved in 35% yield, which is better than the one reported previously (7% yield).^3^ Additionally, the homocoupled product 20 was isolated for the first time here in 21% yield, with the identity of both products confirmed by X-ray analysis.^39^

### Continuous-wave EPR (CW-EPR) of linear bis-nitroxides 1–3, 20

**Fig. 2:**
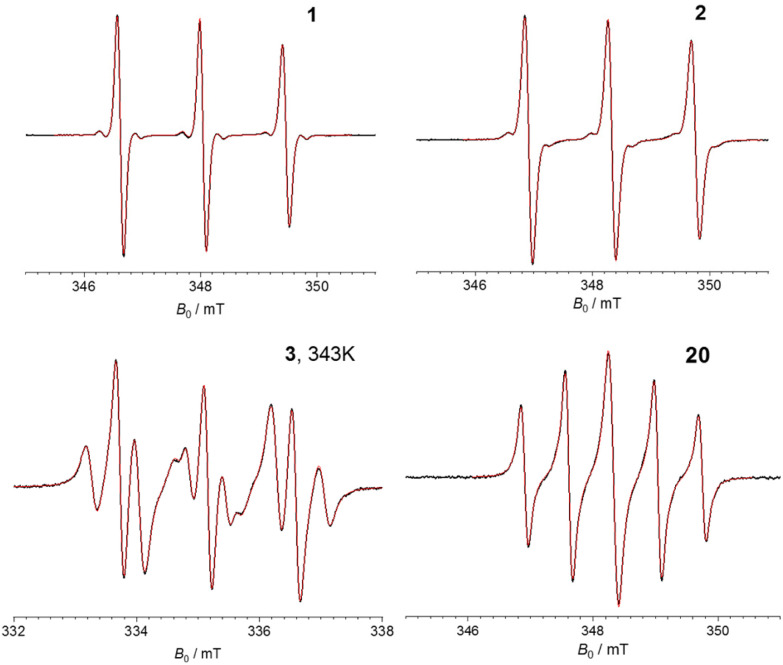
CW-EPR spectra of bis-nitroxides 1–3 and 20 recorded at X-band frequencies (9.7 GHz) at room temperature (298 K), apart from 3 which was measured at 343 K. The black and red lines indicate the experimental and simulated spectra, respectively.

Next, we set out to characterize the electronic communication in the bis-nitroxides 1–3, 20 using continuous-wave EPR (CW-EPR) aiming at resolving and characterizing different spectral features as a result of different exchange coupling. Bis-nitroxide 1 was used as a reference were no exchange coupling should be present as shown previously on 1^3^ and on a Cu^II^–NO^10^ pair, where the Cu^II^ and NO spins where separated by a linker featuring an ester group. Compounds 2, 3 are expected to have (very) small spin–spin communication mediated though the ethyne bond(s). Finally, short bis-ethyne 20 should serve as reference for the two spins showing stronger exchange interaction. The experimental CW-EPR spectra of 1–3, 20 and results of their numerical simulations are presented in Fig. 2. Simulations were performed using software package Orthos^40^ taking into account Brownian rotational diffusion, anisotropic Zeeman, hyperfine and spin–spin dipolar interactions, as well as the isotropic exchange interaction (where applicable) of the two nitroxide fragments.

The CW-EPR spectra of 1–2 exhibit three peaks, characteristic of the NO EPR signal at X-band arising from the hyperfine interaction of the electron spin with the ^14^N nucleus (*A*_N_) with a hyperfine coupling of 39.8 MHz (1.42 mT). Additionally, the ^13^C coupling of 16.8 MHz (0.60 mT) was well resolved for 1 and to a lesser extent for 2. In addition to the diminished ^13^C couplings, the spectrum of 2 is slightly broader compared to that of 1 (by 0.5 MHz, 0.002 mT). These effects could be an indication of a very small *J* in 2 of <1 MHz. However, we cannot exclude that the broadening might originate from a non-infinitely diluted sample or due to the presence of a small amount of oxygen in the sample. In the simulation of 2*J* was not taken into account and the broadening was of Lorentzian shape further suggesting it does not originate from *J*. The CW-EPR spectrum of 3 features a more complicated line splitting where each of the three ^14^N lines is further split, suggesting *J* is comparable to *A*_N_. Indeed, our simulations suggest a *J* of +21.9 MHz (+0.78 mT) and *A*_N_ = 40.1 MHz (1.43 mT), with the features reproduced in three different temperatures (298, 323 and 343 K). The exchange coupling of +21.7 MHz is in agreement with previously published data on 3 ^41^ where *J* was found from X-band CW-EPR at 338 K to be 21.3 ± 0.8 MHz. Here, the positive *J* value indicates the singlet state to be the ground state. The CW-EPR spectrum of 20 shows five lines with a spacing of 19.6 MHz (0.7 mT), *i.e.*, half of the *A*_N_, and the peaks appear with ratio 1 : 1.5 : 1.8 : 1.5 : 1, characteristic of the case where *J* ≫ *A*_N_. This is not surprising considering the presence of the two acetylene groups and the short NO–NO distance (*R*_NO–NO_) of 1.22 nm (from the middle of each NO group) found from X-ray. Simulations of the experimental spectrum suggest a *J* of at least 1100 MHz (40 mT). It should be noted that for 3 and 20 the dipolar coupling was simulated in the point dipolar approximation, with the distance between the NO fragments and their relative orientations taken directly from the solved X-ray structures of the biradicals (Scheme 3c).

**Fig. 3:**
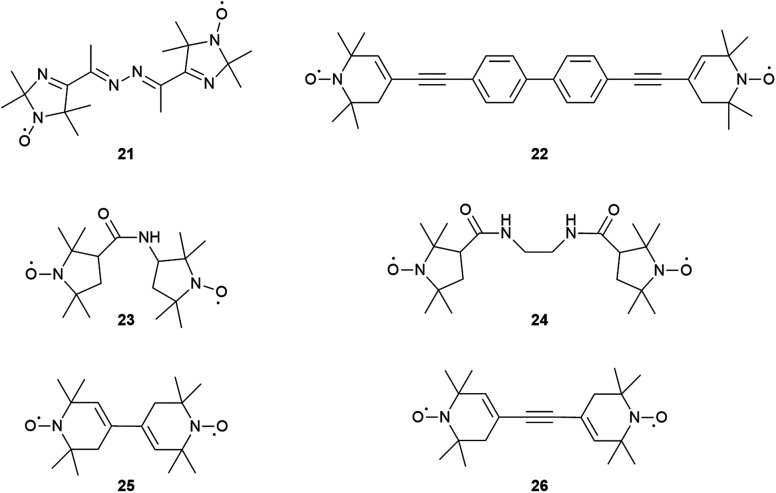
Structure of small bis-nitroxides exhibiting exchange interaction between the paramagnetic nitroxide groups.

The *J* value of 3 is comparable to the one found previously for compound 21 which features a shorter *R*_NO–NO_ and the linker contained two C=N bonds (Fig. 3 and Table 1). A comparison between 3 and 20 makes evident that the biphenyl core reduces *J* by 1100 MHz. Compound 22 is structurally similar to 3 apart from the NO ring and, interestingly, it was found to have a *J* of 12.5 MHz; this suggests the presence of the six-membered NO ring reduced *J* by almost 40% compared to the corresponding five-membered ring. Compound 20 features a larger exchange interaction compared to 23 and 24 having a shorter *R*_NO–NO_ and one or two amide groups, respectively, in the linker between the NO-labelled rings. This suggests that the amide group reduces inter-spin communication compared to the alkyne bond.

**Table 1:** Comparison of the *J* values and NO–NO distances (*R*_NO–NO_) of several bis-nitroxides

Compound	*J*/mT (MHz)	*R*_NO–NO_ a/nm
2	0–0.04 (0–1)^b^	2.04^b^
3	0.77 (21.7)^b^ and ref. 41, 0.43 (12)^3^	2.11^b^^,^^e^, 2.08 (*trans*), 2.07 (*cis*)^41^^, ^c 2.1^3^^,^^c^
20	40 (≥1121)^b^	1.22^b^^,^^e^
21	0.7 (19.6)^43^	1.10^43^^,^^d^^,^^f^
22	0.44 (12.5)^41,44^	2.23^41,44^^,^^d^, 2.33^41,44^^,^^d^
23	30 (>850)^45^	0.88^45^^,^^f^
24	21 (>600)^45^	0.92^45^^,^^f^
25	128 (≥3600)^46^	0.82^46^^,^^f^ 0.85^46^^,^^d^
26	54 (≥1500)^46^	1.10^46^^,^^e^^,^^f^

a Refers to the middle of each N–O bond.

b Presented here.

c Based on geometrical model.

d Based on DFT calculation.

e Based on X-ray structure.

f Based on simulations of rigid limit CW-EPR spectrum.

Compounds 25 and 26 having the six-membered NO ring were found to have a *J* of 3600 and 1500 MHz, respectively. Accounting for the ×0.6 difference between 3 and 22 as discussed above, this means that the bis-nitroxides with the five-membered ring NO groups would be expected to afford *J* values of 5760 and 2400 MHz, therefore allowing a better fine-tuning of *J*. Accurate determination of *J* is dependent not only on the interspin distance, where *J* decays with the distance, but also on the linkage between the spins, the relative orientation of the NO moieties, as well as the nature of the NO-rings. For example it is well established that the ethyne moiety enhances *J*, compared to ethylene or CH_2_ groups due to the increased π orbital overlap of the former and 5-ring nitroxides exhibit larger *J* than 6-ring. The discussion of bis-nitroxides having *J* is not exhaustive; however, it becomes evident that the ‘pool’ of bis-nitroxides and their characterization using CW-EPR techniques^42^ allow us synthesize bis-nitroxides with desirable *J* for use in quantum information processing and DNP experiments.^9^

## Conclusions

In this work we expanded the repertoire of mono- and bis-nitroxide compounds with the NO on the pyrroline ring, we unambiguously characterized the compounds with X-ray crystallography and quantified the *J*-coupling in the bis-nitroxides with CW-EPR techniques. Linear rigid bis-nitroxides 1–3 were readily prepared from the key synthetic precursors TPA 4 and TPC 6 using Sonogashira cross-coupling and Steglich esterification to introduce the nitroxide functionality, respectively. Nitroxide containing aldehyde 5, a key precursor to TPA 4, is most conveniently prepared from reduction of the corresponding Weinreb amide 12. A new method of preparing TPA 4 from aldehyde 5 using a Corey–Fuchs strategy proceeds *via* crystalline vinyl dibromide intermediate 15, which is easier to handle than the alternative vinyl chloride 14. CW-EPR showed diminishing of the exchange coupling in 2 compared to 3, *i.e.* by substituting one triple bond with one ester bond. This suggests one triple bond does not suffice inter-spin communication when the inter-spin distance is in the 2 nm range, even in the case of a fully conjugated system. We also found that removal of the biphenyl core reduces *J* by ∼1 GHz as evidenced form the CW-EPR spectra and respective simulations of 3 and 20. Such observations are important in the design and synthesis of molecules with desired *J* values, typically sought in quantum information processing technologies, as well as in DNP experiments and our findings provide new options for fine-tuning *J* interactions in bis-nitroxide systems.

## Experimental

### General information

Reactions involving moisture- and air-sensitive reagents were carried out in flame-dried glassware under an inert atmosphere (N_2_ or Ar) using standard vacuum line techniques. Anhydrous solvents (CH_2_Cl_2_, Et_2_O, PhMe, THF) were obtained after passing through an alumina column (MBraun SPS-800). Other anhydrous solvents (DMF, DMSO, MeOH) were purchased and used as received. Anhydrous Et_3_N was obtained by distillation from CaH_2_. All other solvents and commercial reagents were used as received without further purification.

Room temperature (rt) refers to 20–25 °C. Temperatures of 0 °C and −78 °C were obtained using ice/water and CO_2(s)_/acetone baths, respectively. Reflux conditions were obtained using DrySyn® blocks equipped with a contact thermometer.

Flash column chromatography purification was performed using silica gel 60 (Merck or Crawford Scientific), alumina gel (Sigma Aldrich) activated with 4% H_2_O or Biotage® IsoleraTM 4, using Biotage® Snap Ultra or Biotage® KP Sil columns (CV = column volume) under the solvent system stated. Analytical thin layer chromatography was performed on pre-coated polystyrene silica TLC sheets (POLYGRAM® SIL G/UV254) or aluminium TLC plates (Merck or Fluorochem). Plates were visualised under UV light (254 nm) or by staining with 1% aq. KMnO_4_ or 6% aq. vanillin followed by heating. Petrol is defined as petroleum ether 40–60 °C.

Melting points (mp) were recorded on an Electrothermal 9100 melting point apparatus and are uncorrected; dec refers to decomposition.

Infrared spectra (*ν*_max_) were recorded on a Shimadzu IRAffinity-1 Fourier transform IR spectrophotometer using either thin film or solid using Pike MIRacle ATR accessory. Analysis was carried out using Shimadzu IRsolution v1.50 and only characteristic peaks are reported. The following abbreviations are used: s, strong; sh, sharp; w, weak; m, medium; br, broad.

^1^H NMR spectra were acquired on either a Bruker Avance 300 (*δ*_H_ 300 MHz), a Bruker Avance II 400 (*δ*_H_ 400 MHz, *δ*_C_ 100 MHz), or a Bruker Ultrashield 500 (*δ*_H_ 500 MHz, *δ*_C_ 126 MHz) spectrometer at ambient temperature in the deuterated solvent stated. The data were analysed using Bruker TopSpin 4.1.1. Chemical shifts, *δ*, are quoted in parts per million (ppm) and are referenced to the residual solvent peak and abbreviation s denotes singlet.

CW-EPR spectra were recorded on deoxygenated samples at room temperature, unless otherwise stated, on a Bruker EMXplus EPR spectrometer with an ELEXSYS super-high-sensitivity probehead (Bruker ER4122SHQE) and nitrogen evaporator variable temperature system (Bruker ER4131VT) operating at ∼9.8 GHz (X-band). All samples were prepared in deuterated PhMe at 500 μM concentration for mono-nitroxides, at 400 μM for 1 and 100 μM for 2, 3 and 20. Samples were contained in 4 mm OD quartz tubes sealed with rubber septa and deoxygenated by saturation with nitrogen or argon gas. For mono-nitroxides the experimental parameters were: modulation amplitude 0.05 mT for 4, 6, 9, 10, 12 and 0.4 mT for 5, 14, 15, time constant and conversion time 20.48 ms for all. The number of scans varied among different samples and DPPH (2,2-diphenyl-1-picrylhydrazyl, *g* = 2.0036) was used to estimate the *g*-values of the mono-radicals. For bis-nitroxides the experimental parameters were: modulation amplitude 0.05 mT for 1, 0.06 mT for 2, 0.01 mT for 3 and 0.08 mT for 20, time constant and conversion time, respectively, 40.96 and 40.96 ms for 1, 2 and 20 and 20.48 and 4.00 ms for 3, number of scans: 1 for 1, 5 for 2 and 20 and 10 for 3.

The simulations of CW EPR spectra of the mono-nitroxides were run with Easyspin^47^ using the ‘garlic’ function assuming fast molecular tumbling and isotropic *g*- and hyperfine-tensors, with electron spin *S* = ½ and considering hyperfine couplings from ^14^N, 2 inequivalent ^1^H (denoted as ^1^H(1), ^1^H(2)) and ^13^C. The number of ^14^N, ^1^H(1), ^1^H(2) and ^13^C nuclei coupled to the electron spin are for all molecules 1, 12, 1, 4, respectively. Additionally, a Voigtian spectral linewidth was added. All parameters were optimized using ‘esfit’ function. For molecules 4, 6, 9, 10, 12 the optimized parameters were obtained from the spectra shown in Fig. S1.† For molecules 5, 14, 15 that are presented with 0.4 mT modulation amplitude (Fig. S1†) an additional spectrum with 0.05 mT modulation amplitude was obtained (data not shown). For these molecules all optimized parameters were obtained from these additional EPR spectra while the ^13^C hyperfine couplings were optimized from the 0.4 mT modulation amplitude EPR spectra. The parameters of the simulated spectra are given in ESI, Table S1.† The simulations of 1, 2, 3 and 20 were performed using software package Orthos.^40^ The theoretical spectra were calculated by solving Stochastic Liouville Equation using the Brownian rotational diffusion as relaxation model. 1 and 2 were simulated as mono-radicals with an isotropic diffusion coefficient (*R*_iso_) of 2.1 × 10^9^ s^−1^ and 2.2 × 10^9^ s^−1^, respectively and a Voigtian spectral lineshape with Gaussian contribution of 0.1 mT for both and Lorentzian contribution of 0.009 mT and 0.046 mT for 1 and 2, respectively. 3 and 20 were simulated as biradicals. For 20 isotropic rotational diffusion with *R*_iso_ = 4.7 × 10^9^ s^−1^ was sufficient to describe the spectra within the experimental uncertainty. For 3 the rotation anisotropy was taken into account by using axial rotation diffusion tensor with *R*_∥_ > 5 × 10^10^ s^−1^ and *R*_⊥_ = 2 × 10^9^ s^−1^. All experimental and simulated spectra are shown after baseline correction and normalization.

Mass spectrometry (*m*/*z*) data were acquired by atmospheric pressure chemical ionisation (APCI) and nanospray ionisation (NSI) at the EPSRC UK National Mass Spectrometry Facility at Swansea University.

Elemental analysis was carried out at the London Metropolitan University where the solid samples were weighed using a Mettler Toledo high-precision scale and analysed using ThermoFlash 2000. The analysis determined the carbon, hydrogen and nitrogen percentage (%CHN). All values are quoted in mass percentage (%).

X-ray diffraction data for all compounds were collected at 173 K using either a Rigaku FR-X Ultrahigh Brilliance Microfocus RA generator/confocal optics with XtaLAB P200 diffractometer [Mo Kα radiation (*λ* = 0.71073 Å)], or a Rigaku MM-007HF High Brilliance RA generator/confocal optics with XtaLAB P100 diffractometer [Cu Kα radiation (*λ* = 1.54187 Å)]. Intensity data were collected using *ω* steps or both *ω* and *φ* steps accumulating area detector images spanning at least a hemisphere of reciprocal space. Data were collected and processed (including correction for Lorentz, polarization and absorption) using CrystalClear.^48^ Structures were solved by charge-flipping (Superflip^49^), direct (SIR-2011^50^), dual-space (SHELXT^51^) or Patterson methods (PATTY^52^) and refined by full-matrix least-squares against *F*^2^ (SHELXL-2018/3^53^). Non-hydrogen atoms were refined anisotropically, and hydrogen atoms were refined using a riding model. All calculations were performed using the Olex2^54^ interface. CCDC 2212061–2212068 contains the supplementary crystallographic data for this paper.†

The research data supporting this publication can be accessed at https://doi.org/10.17630/75719a96-4ec5-4e45-a838-7f34642d4151.^55^

### Compound data

#### 2,2,5,5-Tetramethyl-2,5-dihydro-1*H*-pyrrole-3-carboxylic acid amide (8)^56^

Following a literature procedure,^14,16,30,31^ a solution of 2,2,6,6-tetramethyl-4 piperidone 7 (12.0 g, 77.4 mmol, 1.0 equiv.) in glacial AcOH (48 mL) was cooled to 0 °C before a solution of Br_2_ (8 mL, 156 mmol, 2.0 equiv.) in glacial AcOH (34 mL) was added dropwise over the course of 6 h. The reaction was stirred at rt for 24 h before the resulting suspension was filtered, and the precipitate washed successively with glacial AcOH (30 mL), H_2_O (30 mL) and Et_2_O (2 × 30 mL) to give 3,5-dibromo-2,2,6,6-tetramethyl-4-oxopiperidine hydrobromide (22.8 g) as a beige solid that was used without further purification.

Intermediate dibromide (22.8 g) was suspended in 35% aq. NH_3_ (70 mL) and stirred at rt for 2 h. The solution was filtered, and the filtrate was saturated with KOH pellets until pH ∼ 14 was obtained. The resulting suspension was filtered and concentrated under reduced pressure to give the crude product, which was purified by recrystallisation from PhMe to give 8 (7.5 g, 44.7 mmol, 58% yield (over two steps)) as brown crystals. mp 177–178 {Lit.^56^ 179–180 °C}; *v*_max_ (cm^−1^) 1359s, 1598s, 1650s, 1662s, 2962br, 3178–3346br; ^1^H NMR (400 MHz, CDCl_3_) *δ*_H_: 1.29 (6 H, s, C*H*_3_), 1.45 (6 H, s, C*H*_3_), 6.17 (1 H, s, C=C*H*); ^13^C NMR (100 MHz, CDCl_3_) *δ*_C_: 30.2 (2 × *C*H_3_), 30.3 (2 × *C*H_3_), 63.5 (*C*NH), 66.9 (*C*NH), 68.9 (C*C*HC), 142.3 (*C*CO), 166.0 (*C*O).

#### 1-Oxyl-2,2,5,5-tetramethyl-2,5-dihydro-1*H*-pyrrole-3-carboxamide (9)^31^

Amide 8 (5.0 g, 30 mmol, 1.0 equiv.) was dissolved in H_2_O (55 mL) at rt before EDTA (0.3 g, 0.9 mmol, 3.1 mol%) and Na_2_WO_4_·2H_2_O, (0.3 g, 0.85 mmol, 2.9 mol%) were added and the solution stirred until all salts were dissolved. A solution of 30% w/w H_2_O_2_ (5.5 mL, 183 mmol, 6.1 equiv.) was added slowly and the reaction was stirred at rt in the dark for 5 min before being placed in a fridge (4–8 °C) and left to stand for 5 days. The resulting suspension was filtered and the solid rinsed with H_2_O to give 9 (4.0 g, 22 mmol, 73%) as yellow crystals. mp 173–175 °C {Lit.^31^ 174.0–174.5 °C}; *v*_max_ (cm^−1^) 783w, 1161w, 1188w, 1363w, 1595m, 1635s, 2322br, 3188br, 3367br; *m*/*z* (APCI^+^) 184 ([*M* + H]^+^, 100%); HRMS (APCI^+^) C_9_H_17_N_2_O ([*M* + H]^+^) found 185.1282, calculated 185.1285 (−1.4 ppm).

#### 1-Oxyl-2,2,5,5-tetramethyl-2,5-dihydro-1*H*-pyrrole-3-carboxylic acid (TPC, 6)^16^

A solution of 9 (4.0 g, 22 mmol, 1.0 equiv.) in 10% w/v NaOH (100 mL) was heated to 100 °C for 2 h. The solution was acidified with 6 M HCl to pH ∼ 1 and the resulting precipitate was filtered and dried. The filtrate was washed with Et_2_O (×3), before the combined organic phases were dried over MgSO_4_ and concentrated under reduced pressure. The resulting solid was combined with the dried precipitate to give TPC 6 (2.1 g, 11.4 mmol, 52%) as yellow crystals. mp 210 °C (dec) {Lit.^31^ 210–211 °C (dec)}; *v*_max_ (cm^−1^) 765sh, 1037sh, 1155s, 1188s, 1276m, 1404w, 1720s, 2330w, 3100br; *m*/*z* (APCI^+^) 479 ([*M* + H]^+^, 100%); HRMS (APCI^+^) C_9_H_15_NO_3_ ([*M* + H]^+^) found 185.1045 calculated 185.1046 (−0.8 ppm), 478 ([*M*]^+^, 100%); HRMS (APCI^+^) C_9_H_14_NO_3_ ([*M*]^+^) found 184.0970, calculated 184.0968 (+1.0 ppm).

#### 3-(Hydroxymethyl)-2,2,5,5-tetramethyl-2,5-dihydro-1*H*-pyrrol-1-oxyl (10)^16^

Red-Al® (12.0 mL, 39.0 mmol, 3.6 equiv.) was added dropwise over the course of 5 min to a solution of TPC 6 (2.0 g, 10.8 mmol, 1.0 equiv.) in anhydrous PhMe (35 mL) under an atmosphere of Ar at rt. The reaction was then heated to 55 °C for 90 min before being left to cool to rt and being quenched with 5% w/v NaOH. The phases were separated, and the aqueous extracted with PhMe (4 × 30 mL). The combined organics were dried over MgSO_4_ and concentrated under reduced pressure to give 10 (1.6 g, 9.4 mmol, 87%) as yellow crystals. mp 75–77 °C {Lit.^57^ 75–77 °C}; *v*_max_ (cm^−1^) 856s, 1040s, 1111w, 1161s, 1224w, 1278s, 1359m, 1460m, 2978m, 3371br; *m*/*z* (NSI^+^) 171 ([*M* + H]^+^, 100%); HRMS (NSI^+^) C_9_H_17_NO_2_ ([*M* + H]^+^) found 171.1250, calculated 171.1254 (−2.2 ppm).

#### *N*-Methoxy-*N*,2,2,5,5-pentamethyl-2,5-dihydro-1*H*-pyrrole-3-carboxamide (11)^30^

Following a literature procedure,^14,30^ a solution of 2,2,6,6-tetramethyl-4 piperidone 7 (15.0 g, 96.6 mmol, 1.0 equiv.) in glacial AcOH (60 mL) was cooled to 0 °C before a solution of Br_2_ (10 mL, 195 mmol, 2.0 equiv.) in glacial AcOH (45 mL) was added dropwise over the course of 6 h. The reaction was stirred at rt overnight before the resulting suspension was filtered, and the precipitate washed successively with glacial AcOH (100 mL), H_2_O (2 × 100 mL) and Et_2_O (2 × 100 mL) to give 3,5-dibromo-2,2,6,6-tetramethyl-4-oxopiperidine hydrobromide (24.7 g) as a beige solid that was used without further purification.

A solution of *N*,*O*-dimethylhydroxylamine hydrochloride (9.9 g, 102 mmol, 1.2 equiv.) in H_2_O (58 mL) was cooled to 0 °C before NEt_3_ (49 mL, 352 mmol, 4.1 equiv.) was added. The dibromide intermediate (33.5 g, 85 mmol, 1.0 equiv.) was then added in portions over the course of 7 h at rt. The reaction was stirred at rt for 7 days. The pH was adjusted to between 9.0 and 9.5 using 40% w/v aq. NaOH and the solution extracted with EtOAc (3 × 60 mL). The combined organic phases were dried over Na_2_SO_4_ and concentrated under reduced pressure. Product 11 (13.0 g, 62 mmol, 65% over two steps) was pure by TLC analysis and was used without further purification as a light orange oil. Upon multiple repeats of the reaction the crude product was not as pure, but could be purified by flash column chromatography on silica gel (CH_2_Cl_2_/MeOH 98 : 2 to 90 : 10). *v*_max_ (cm^−1^) 750sh, 972m, 1178m, 1352s, 1610m, 1654m, 2962w; ^1^H NMR (500 MHz, CDCl_3_) *δ*_H_: 1.24 (6 H, s, 2 × C*H*_3_), 1.34 (6 H, s, 2 × C*H*_3_), 3.16 (3 H, s, NC*H*_3_), 3.56 (3 H, s, OC*H*_3_), 6.04 (1 H, s, CC*H*_vin_C); *m*/*z* (NSI^+^) 213 ([*M* + H]^+^, 100%); HRMS (NSI^+^) C_11_H_21_N_2_O_2_ ([*M* + H]^+^) found 213.1594, calculated 213.1598 (−1.7 ppm).

#### 1-Oxyl-*N*-methoxy-*N*,2,2,5,5-pentamethyl-2,5-dihydro-1*H*-pyrrole-3-carboxamide (12)^30^

Amide 11 (32.1 g, 14.8 mmol, 1 equiv.) was dissolved in H_2_O (27 mL) at rt before EDTA (0.14 g, 0.47 mmol, 2.1 mol%), Na_2_WO_4_·2H_2_O (0.14 g, 0.42 mmol, 2.4 mol%) and 30% w/v aq. H_2_O_2_ (2.8 mL, 91 mmol, 6.1 equiv.) were added. The reaction was stirred for 8 min at rt before being left to stand in the dark at rt for 2 d. The pH was adjusted to between 5.0 and 6.0 with conc. HCl and the aqueous extracted with EtOAc (3 × 50 mL). The combined organic phases were dried over Na_2_SO_4_ and concentrated under reduced pressure to give 12 (2.5 g, 11.1 mmol, 75%) as bright orange crystals. mp 58–60 °C; *v*_max_ (cm^−1^) 746sh, 842sh, 972m, 1161m, 1274w, 1357s, 1365s, 1427m, 1463m, 1610s, 1648s, 2931w, 2974 m; *m*/*z* (NSI^+^) 228 ([*M* + H]^+^, 100%); HRMS (NSI^+^) C_11_H_20_N_2_O_3_ ([*M* + H]^+^) found 228.1467, calculated 228.1468 (−0.6 ppm).

#### 1-Oxyl-2,2,5,5-tetramethyl-2,5-dihydro-1*H*-pyrrole-3-carbaldehyde (5)^14,16,30^

##### From 10

A solution of oxalyl chloride (0.25 mL, 2.6 mmol, 1.1 equiv.) in CH_2_Cl_2_ (3 mL) was cooled to −60 °C under an atmosphere of N_2_ before a solution of anhydrous DMSO (0.4 mL, 5.6 mmol) in CH_2_Cl_2_ (3 mL) was added dropwise over the course of 7 min. The reaction mixture was stirred for 3 min before a solution of 10 (0.41 g, 2.4 mmol, 1.0) in CH_2_Cl_2_ was added. The reaction was stirred at −60 °C for 15 min before NEt_3_ (2 mL, 14 mmol, 5.4 equiv.) was added and the solution was stirred for 5 min. The solution was allowed to warm to rt before being quenched with H_2_O (25 mL). The phases were separated and the aqueous extracted with CH_2_Cl_2_. The combined organic phases were washed with brine (20 mL), 1% w/w sulfuric acid (9 mL), H_2_O (9 mL), and 5% w/v NaHCO_3_ (9 mL), dried over MgSO_4_ and concentrated under reduced pressure. The crude product was purified by sublimation at 50 °C to give 5 (0.23 g, 1.34 mmol, 56%) as yellow crystals. mp 76–78 °C (dec) {Lit.^30^ 77–79 °C}; *v*_max_ (cm^−1^) 765sh, 869sh, 952w, 1116w, 1161w, 1278m, 1350m, 1622w, 1680s; 2978w; *m*/*z* (APCI^+^) 168 ([*M*]^+^, 100%); HRMS (APCI^+^) C_9_H_14_NO_2_ ([*M*]^+^) found 168.1016, calculated 168.1019 (−1.8 ppm).

##### From 12

A solution of 12 (3.9 g, 17.4 mmol, 1.0 equiv.) in Et_2_O (22 mL) was cooled to −78 °C under an atmosphere of N_2_ before a solution of DIBAL-H (18.0 mL, 21.6 mmol, 1.2 m in PhMe, 1.2 equiv.) was added dropwise over the course of 30 min. After stirring for 15 min, the reaction was quenched by slowly pouring the mixture into a solution of aq. 2 M HCl (22.0 mL) at 0 °C. The mixture was allowed to warm to rt and the phases separated. The aqueous was extracted with EtOAc (3 × 30 mL), and the combined organics were dried over MgSO_4_ and concentrated under reduced pressure to give 5 (2.8 g, 16.6 mmol, 95%) as yellow crystals. The product was used for the next step without further purification. Data as above.

#### 3-(2-Chlorovinyl)-2,2,5,5-tetramethyl-2,5-dihydro-1*H*-pyrrol-1-oxyl (14)^14,16^

Chloromethyl triphenylphosphonium chloride 13 (11.5 g. 33.0 mmol, 4.0 equiv.) was suspended in THF (80 mL) under an atmosphere of N_2_ at −78 °C before *n*-BuLi (11.1 mL, 29.6 mmol, 2.5 M in hexanes, 3.6 equiv.) was added dropwise over the course of 30 min. The temperature was increased to −40 °C and the solution stirred for 30 min. The solution was then cooled to −78 °C before a solution of 5 (1.4 g, 8.3 mmol, 1.0 equiv.) in THF (20 mL) was added dropwise over the course of 20 min. The dry ice-acetone bath was replaced by an ice-water bath and the solution was stirred at 0 °C for 30 min before being quenched with ice-cold H_2_O (8 mL). The passes were separated and the organics were removed under reduced pressure before H_2_O (20 mL) was added and the combined aqueous phases are extracted with EtOAc (3 × 30 mL). The combined organic phases were dried over Na_2_SO_4_ and concentrated under reduced pressure. The crude product was suspended in hexane and the undissolved solid removed by filtration. The filtrate was concentrated under reduced pressure and the resulting orange oil purified by flash column chromatography on silica gel (CH_2_Cl_2_ : MeOH 10 : 0 to 9 : 1) to give 14 (1.63 g, 8.1 mmol, 98%) as an orange oil. *v*_max_ (cm^−1^) 626s, 694m, 740m, 844s, 941s, 1160s, 1224, 1284w, 1357s, 1465m, 1610w, 2929w, 2974s; *m*/*z* (APCI^+^) 201 ([*M* + H]^+^, 100%); HRMS (APCI^+^) C_10_H_16_NOCl ([*M* + H]^+^) found 201.0911, calculated 201.0915 (−2.0 ppm).

#### 3-(2,2-Dibromovinyl)-2,2,5,5-tetramethyl-2,5-dihydro-1*H*-pyrrol-1-oxyl (15)

CBr_4_ (4.6 g, 14 mmol, 1.9 equiv.) was dissolved in CH_2_Cl_2_ (60 mL) under an atmosphere of N_2_ at 0 °C before a solution of PPh_3_ (7.3 g, 28 mmol, 3.9 equiv.) in CH_2_Cl_2_ (30 mL) was added dropwise over the course of 10 min. The mixture was stirred at 0 °C for 30 min before a solution of 5 (1.2 g, 7.0 mmol, 1.0 equiv.) in CH_2_Cl_2_ (15 mL) was added dropwise over the course of 10 min. The reaction was stirred at 0 °C for 30 min, allowed to warm to rt and stirred for 1.5 h before being quenched with H_2_O (30 mL). The phases were separated, the aqueous extracted with CH_2_Cl_2_ (2 × 20 mL), and the combined organics were washed with NaHCO_3_ (20 mL), brine (20 mL), dried over MgSO_4_ and concentrated under reduced pressure. The crude product was purified by flash column chromatography on silica gel (CH_2_Cl_2_) to give 15 (1.5 g, 4.6 mmol, 66%) as orange solid. Crystals suitable for X-ray analysis were obtained after recrystallisation from hexane. mp 67–68 °C; *v*_max_ (cm^−1^) 767m, 840s, 871s, 883s, 945m, 1161s, 1224br, 1269br, 1429w, 1456w, 1579m, 2970w; *m*/*z* (NSI^+^) 322 ([*M* + H]^+^, 100%); HRMS (NSI^+^) C_10_H_15_Br_2_NO ([*M* + H]^+^) found 322.9512, calculated 322.9515 (−0.9 ppm).

#### 3-Ethynyl-2,2,5,5-tetramethyl-2,5-dihydro-1*H*-pyrrol-1-oxyl (TPA, 4)^14,16^

##### From 14

A solution of 14 (0.15 g, 0.7 mmol, 1.0 equiv.) in THF (6 mL) was subjected to three freeze–pump–thaw cycles. KO*t*-Bu (0.5 g, 4.1 mmol, 5.8 equiv.) was added at rt before the reaction was heated to 55 °C for 2 h. The solution was allowed to cool to rt before being quenched with aqueous NH_4_Cl (0.3 mL, 1 M) and dried under reduced pressure. The dried product was re-dissolved in Et_2_O (8 mL) and washed with H_2_O (3 × 4 mL) and brine (4 mL), and the organic phase dried over MgSO_4_ and concentrated under reduced pressure. The crude product was purified by sublimation (51 °C) to give TPA 4 (0.03 g, 0.17 mmol, 26%) as yellow solid. mp 122–123 °C {Lit.^15^ 122–123 °C}; *v*_max_ (cm^−1^) 752s, 879s, 1087w, 1159s, 1274m, 1359s, 1438m, 1456m, 2092w, 2933w 2980s, 3194s; *m*/*z* (APCI^+^) 164 ([*M*]^+^, 100%); HRMS (APCI^+^) C_10_H_14_NO ([*M*]^+^) found 164.1066, calculated 164.1070 (−2.4 ppm).

##### From 15

A solution of 15 (0.47 g, 1.5 mmol, 1.0 equiv.) in THF (20 mL) was cooled to −78 °C under an atmosphere of N_2_ before *n*-BuLi (1.5 mL, 2.9 mmol, 1.9 m in hexanes, 1.9 equiv.) was added dropwise over the course of 5 min. The reaction was stirred at −78 °C for 45 min before being allowed to warm to rt and stirred for a further 3 h, followed by quenching with H_2_O (20 mL). The phases were separated. The aqueous was extracted with Et_2_O (3 × 20 mL), and the combined organics dried over Na_2_SO_4_ and concentrated under reduced pressure. The crude product was purified by flash column chromatography on silica gel (CH_2_Cl_2_) to give TPA 4 (0.023 g, 0.14 mmol, 10%). Data as above.

#### Bis(1-oxyl-2,2,5,5-tetramethyl-2,5-dihydro-1*H*-pyrrol-3-yl) [1,1′-biphenyl]-4,4′-dicarboxylate (1)^32^

As previously reported,^32^ 4,4-biphenol 16 (0.10 g, 0.54 mmol, 1 equiv.), TPC 6 (0.30 g, 1.63 mmol, 3 equiv.), and DMAP (0.20 g, 1.64 mmol, 3 equiv.) were dissolved in THF (10 mL) under an N_2_ atmosphere. The flask was covered in tin foil before EDCI·HCl (0.16 g, 1.03 mmol, 2 equiv.) was added and the reaction stirred at rt for 24 h. The solution was filtered to remove the urea precipitate, followed by washing the solid with CH_2_Cl_2_. The organic filtrate was washed with H_2_O (×3), dried over MgSO_4_ and concentrated under reduced pressure. The crude product was purified by flash column chromatography on alumina gel (4% H_2_O, CH_2_Cl_2_) to give 1 (0.23 g, 0.44 mmol, 82%) as yellow crystals. mp 211–213 °C; *v*_max_ (cm^−1^) 2976w, 2931w, 1732s, 1490m, 1346m, 1286m, 1244m, 1194s, 1147s; HRMS (NSI^+^) C_30_H_35_N_2_O_6_ ([M + H]^+^), found 519.2476, calculated 519.2490 (−1.4 ppm). C_30_H_34_N_2_O_6_ calculated C, 69.48; H, 6.61; N, 5.40%; found C, 69.30; H, 6.75; N, 5.54%.

#### 4′-Iodo-[1,1′-biphenyl]-4-yl-1-oxyl-2,2,5,5-tetramethyl-2,5-dihydro-1*H*-pyrrole-3-carboxylate (18)

Prepared as previously reported.^37^ The data for 18 are identical to those previously reported in ref. 37.

#### 4′-((1-Oxyl-2,2,5,5-tetramethyl-2,5-dihydro-1*H*-pyrrol-3-yl)ethynyl)-[1,1′-biphenyl]-4-yl 1-oxyl-2,2,5,5-tetramethyl-2,5-dihydro-1*H*-pyrrole-3-carboxylate (2)

PdCl_2_(PPh_3_)_2_ (0.012 g, 0.018 mmol, 25 mol%) and 18 (0.033 g, 0.071 mmol, 1 equiv.) were dissolved in NEt_3_ (12 mL) under an atmosphere of N_2_ at rt. DMF (1 mL) was added to the mixture to increase solubility. In a second flask, TPA 4 (0.022 g, 0.13 mmol, 1.8 equiv.) and PPh_3_ (0.0132 g, 0.05 mmol, 0.7 equiv.) were dissolved in NEt_3_ (5 mL) and DMF (1 mL). The two solutions were degassed by freeze–pump–thaw cycles (×3), before CuI (0.011 g, 0.06 mmol, 0.85 equiv.) was added to the solution of 18, followed by dropwise addition of the second solution containing TPA 4. The reaction was stirred at rt for 3 d before the solvents were removed under reduced pressure, the residue dissolved in CH_2_Cl_2_ (30 mL) and washed with H_2_O (3 × 30 mL). The organic phase was dried over Na_2_SO_4_ and concentrated under reduced pressure. The crude product was purified by flash column chromatography on silica gel (petrol/EtOAc 60 : 40) to give 2 (0.01 g, 0.021 mmol, 28%) as yellow solid. Crystals suitable for X-ray analysis were obtained after recrystallisation from CHCl_3_. mp 230–232 °C (dec); *v*_max_ (cm^−1^) 759m, 800s, 840m, 875m, 1000sh, 1149s, 1246w, 1286m, 1384m, 1446w, 1490n, 1732s, 2978w; *m*/*z* (APCI^+^) 499 ([*M* + H]^+^, 100%); HRMS (APCI^+^) C_31_H_35_N_2_O_4_ ([*M* + H]^+^) found 499.2585, calculated 499.2591 (−1.3 ppm); C_31_H_34_N_2_O_4_ calculated C, 74.67; H, 6.87; N, 5.62%; found C, 74.67; H, 6.95; N, 5.51%.

#### 3,3′-([1,1′-Biphenyl]-4,4′-diylbis(ethyne-2,1-diyl))bis(2,2,5,5-tetramethyl-2,5-dihydro-1*H*-pyrrol-1-oxyl) (3)^3^

Following a modified literature procedure,^16^ PdCl_2_(PPh_3_)_2_ (0.025 g, 0.036 mmol, 30 mol%) and 4,4′-diiodobiphenyl 19 (0.048 g, 0.12 mmol, 1.0 equiv.) were dissolved NEt_3_ (15 mL) and DMF (3 mL) under an atmosphere of N_2_ at rt. In a second flask, TPA 4 (0.040 g, 0.24 mmol, 2 equiv.) and PPh_3_ (0.022 g, 0.082 mmol, 0.68 equiv.) were dissolved in NEt_3_ (5 mL) and DMF (2 mL). The two solutions were degassed by freeze–pump–thaw cycles (×3), before CuI (0.027 g, 0.14 mmol, 26 mol%) was added to the solution of 19 followed by dropwise addition of the second solution containing TPA 4. The reaction was stirred at rt for 3 d, before the solvents were removed under reduced pressure, the residue dissolved in CH_2_Cl_2_ (30 mL) and washed with H_2_O (3 × 40 mL) and brine (30 mL). The organic phase was dried over MgSO_4_ and concentrated under reduced pressure. The crude product was purified by Biotage® Isolera™ 4 [SNAP KP-Sil 10 g, 36 mLmin^−1^, hexane/EtOAc (100 : 0 to 85 : 15 12 CV, 85 : 15 7 CV, 75 : 25 3 CV, 65 : 35 5 CV, 50 : 50 5CV)] to give 3 (0.02 g, 0.04 mmol, 35%) as beige crystalline solid. Crystals suitable for X-ray analysis were obtained after recrystallisation from hexane/EtOAc (1 : 1). mp 237–238 °C (dec); *v*_max_ (cm^−1^) 759w, 820s, 881w, 1002w, 1163m, 1247m, 1357m, 1462w, 2850w, 2922m, 2976w, 3051w; *m*/*z* (APCI^+^) 479 ([*M* + H]^+^, 100%); HRMS (APCI^+^) C_32_H_35_N_2_O_2_ ([*M* + H]^+^) found 479.2689, calculated 479.2693 (−0.8 ppm); C_32_H_34_N_2_O_2_ calculated C, 80.3; H, 7.16; N, 5.85%; found C, 80.42; H, 7.02; N, 5.87%.

A second fraction containing 3,3′-(buta-1,3-diyne-1,4-diyl)bis(2,2,5,5-tetramethyl-2,5-dihydro-1*H*-pyrrol-1-oxyl) 20 (0.08 g, 0.024 mmol, 21%) was also obtained as a yellow solid. Crystals suitable for X-ray analysis were obtained after recrystallisation from petrol/EtOAc (60 : 40). mp 225–227 °C (dec); *v*_max_ (cm^−1^) 885w, 997w, 1093s, 1155m, 1438s, 1479m, 2320br; *m*/*z* (NSI^+^) 327 ([M + H]^+^, 100%); HRMS (APCI^+^) C_20_H_27_N_2_O_2_ ([M + H]^+^) found 327.2074, calculated 327.2072 (+0.6 ppm).

## Conflicts of interest

There are no conflicts of interest to declare.

## Supplementary Material

OB-021-D2OB01863B-s001

OB-021-D2OB01863B-s002
