# Supplementary material for: Synthesis of mono-nitroxides and of bis-nitroxides with varying electronic through-bond communication
Source: Org Biomol Chem. 2022 Dec 8;21(2):375–85. doi: 10.1039/d2ob01863b (PMC9811921; doi:10.1039/d2ob01863b)
Supplement: OB-021-D2OB01863B-s001 [file OB-021-D2OB01863B-s001.pdf]

Electronic Supplementary Material (ESI) for Organic & Biomolecular Chemistry.
This journal is © The Royal Society of Chemistry 2022

Supporting Information

Synthesis of mono-nitroxides and of bis-nitroxides with varying electronic through-bond communication

Angeliki Giannoulis,*[,a,b] Katrin Ackermann,[b] Alexey Bogdanov,[a] David B. Cordes,[b] Catherine Higgins,[b] Joshua Ward,[b] Alexandra M. Z. Slawin,[b] James E. Taylor*[,b,c] and Bela E. Bode*[,b]

[a]*Department of Chemical and Biological Physics, Weizmann Institute of Science, Rehovot, 76100, Israel.*

[b]*EaStCHEM School of Chemistry, Biomedical Sciences Research Complex and Centre of Magnetic Resonance, University of St Andrews, North Haugh, St Andrews, KY16 9ST, U.K.*

[c]*Department of Chemistry, University of Bath, Claverton Down, Bath, BA2 7AY, U.K.*

E-mail: beb2@st-andrews.ac.uk; angeliki.giannoulis@weizmann.ac.il; j.e.taylor@bath.ac.uk

Additional CW-EPR spectra

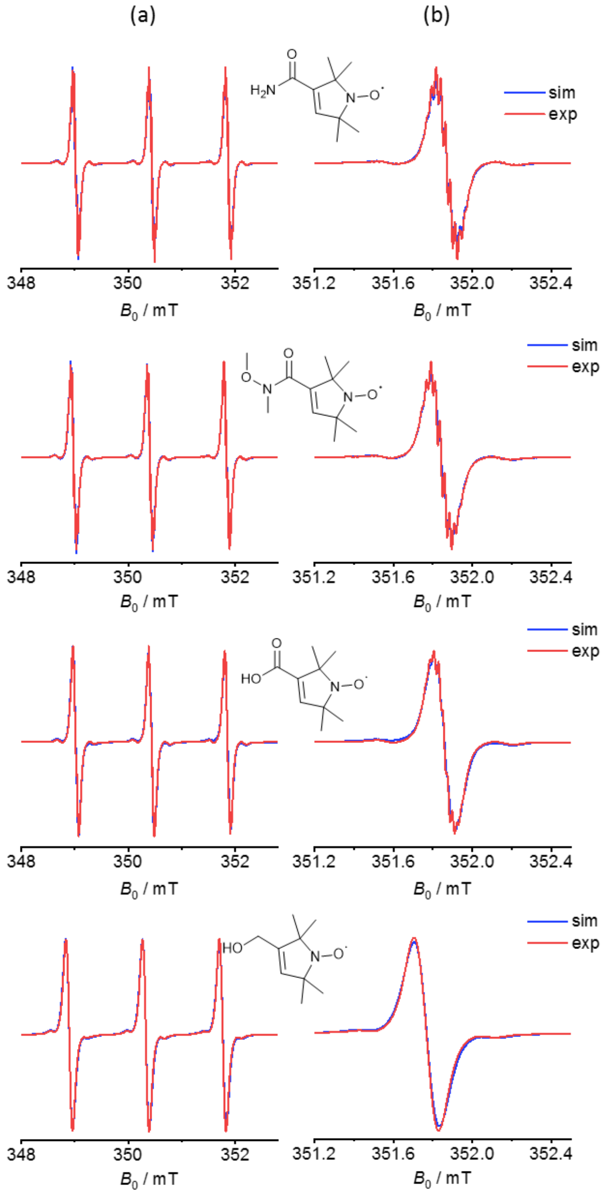

Figure S1 CW-EPR of mono-radicals recorded at room temperature at X-band (a) full spectrum and (b) zoom-in the high field ^{14}N line with the experiment and simulation in red and blue colour, respectively. Simulations were run with Easyspin and simulation parameters are given in **Table S1**.

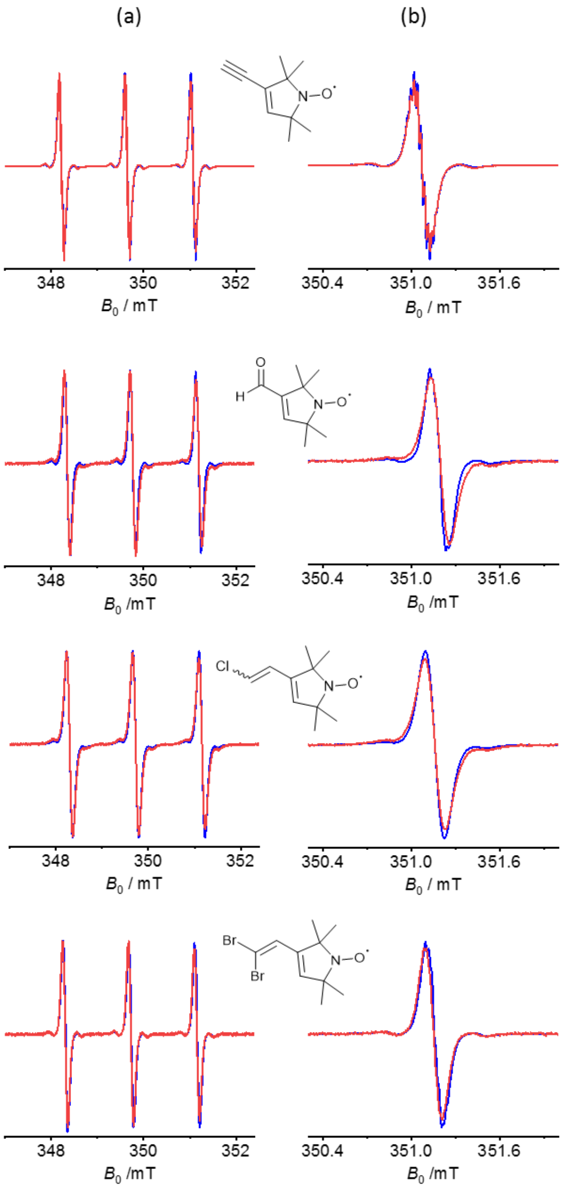

Figure S1 continued CW-EPR of mono-radicals recorded at room temperature at X-band (a) full spectrum and (b) zoom-in the high field ^{14}N line with the experiment and simulation in red and blue colour, respectively. Simulations were run with Easyspin and simulation parameters are given in **Table S1**.

Table S1 Parameters used for the simulations of the mono-radicals.

	g-value	$A_{^{14}N}$ / MHz	$A_{^{1}H(1)}$ / MHz	$A_{^{1}H(2)}$ / MHz	$A_{^{13}C}$ / MHz	linewidth / Voigtian
**4**	2.01049	40.044	0.655	1.31	17.45	0.010, 0.035
**5**	2.00776	39.95	0.66	1.32	16.75	0.007 0.037
**6**	2.00393	39.8	0.625	1.25	16.75	0.00, 0.047
**9**	2.00594	40.044	0.655	1.3	16.75	0.017, 0.025
**10**	2.00658	40.3	0.625	1.25	16.75	0.00, 0.09
**12**	2.00613	40.19	0.645	1.29	16.75	0.00, 0.04
**14**	2.00901	40.25	0.685	1.37	16.75	0.013, 0.075
**15**	2.00898	40.090	0.65	1.3	17.13	0.005, 0.034

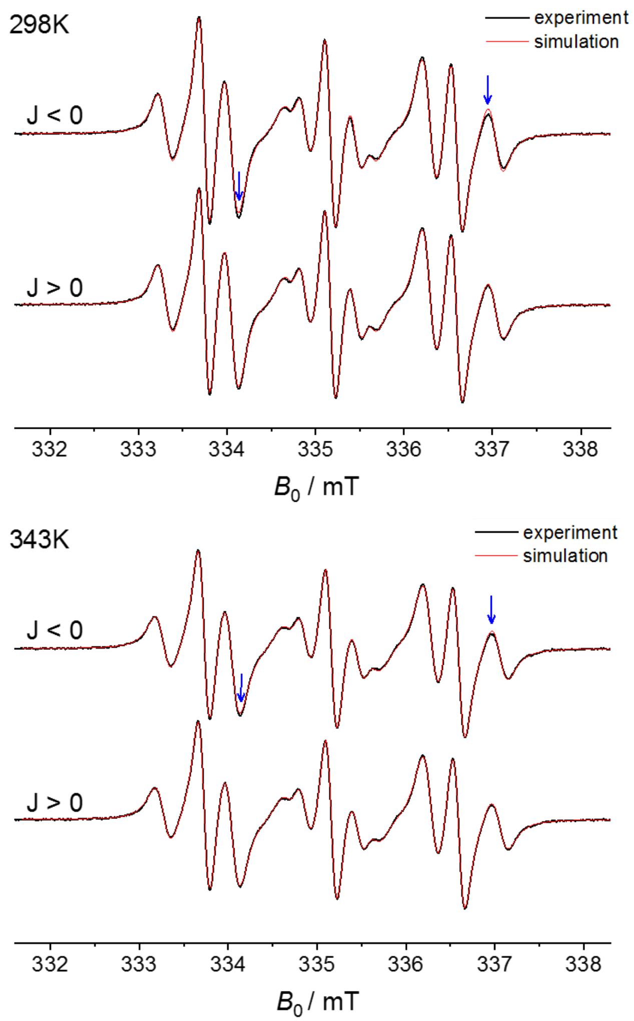

Figure S2 CW-EPR of **3** recorded at room temperature (298 K) and at 343 K at X-band along with simulations in black and red colour, respectively. Simulations were run with positive and negative value of *J* using a self-written Matlab script with the simulation parameters given in main text. The blue arrows denote the deviation of the simulation from the experimental spectrum, suggesting a $J > 0$. Parameters of experiment: 9.48 GHz, 23 dB attenuation, modulation amplitude 0.01 mT, 10 scans.

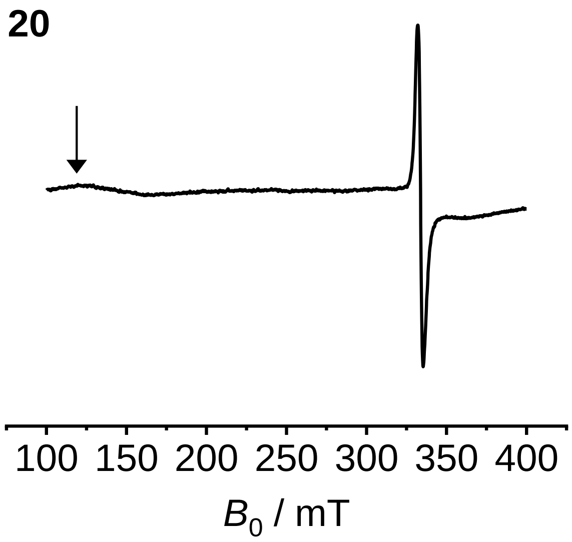

Figure S3 CW-EPR of **20** recorded at 5 K with the arrow indicating the half-field transition. Parameters of experiment: 9.346GHz, 13 dB attenuation, modulation amplitude 0.2 mT, 1 scan.

Characterization spectra

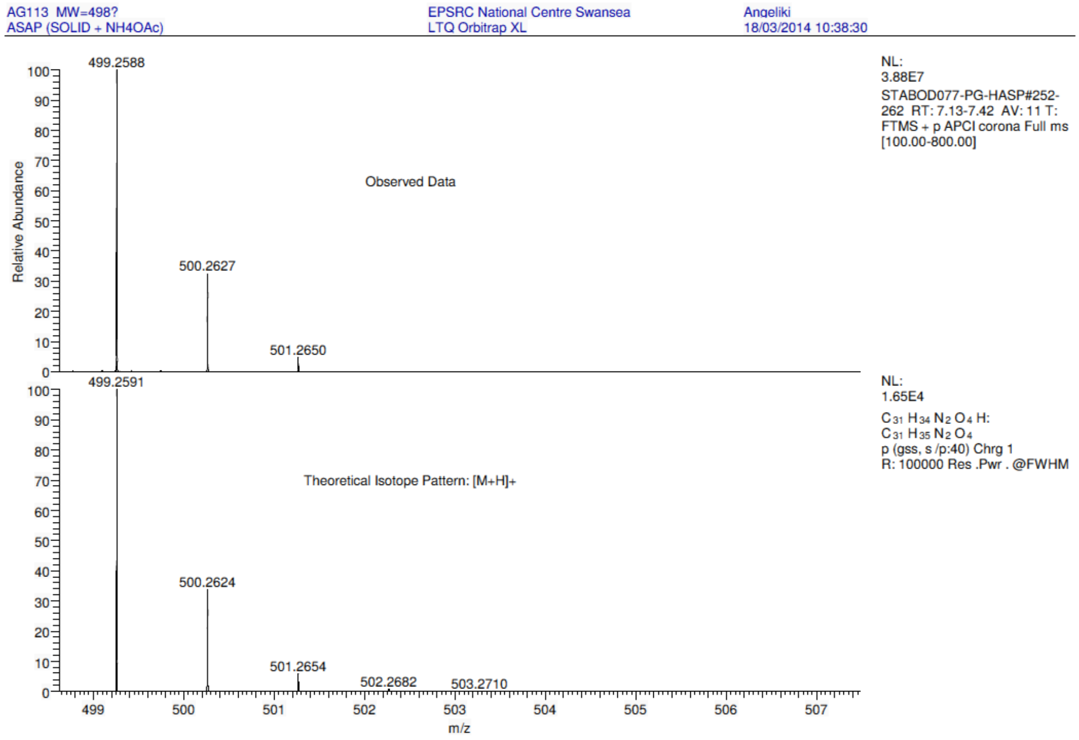

Figure S4 Mass spectrum of **2**.

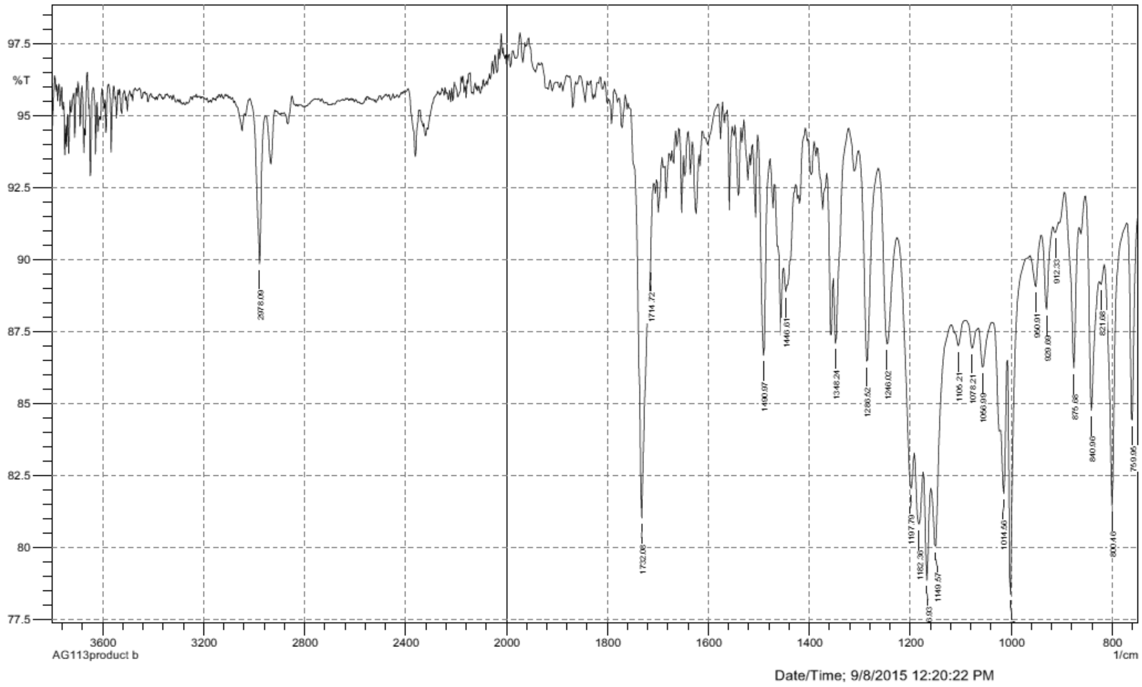

Figure S5 IR spectrum of **2.**

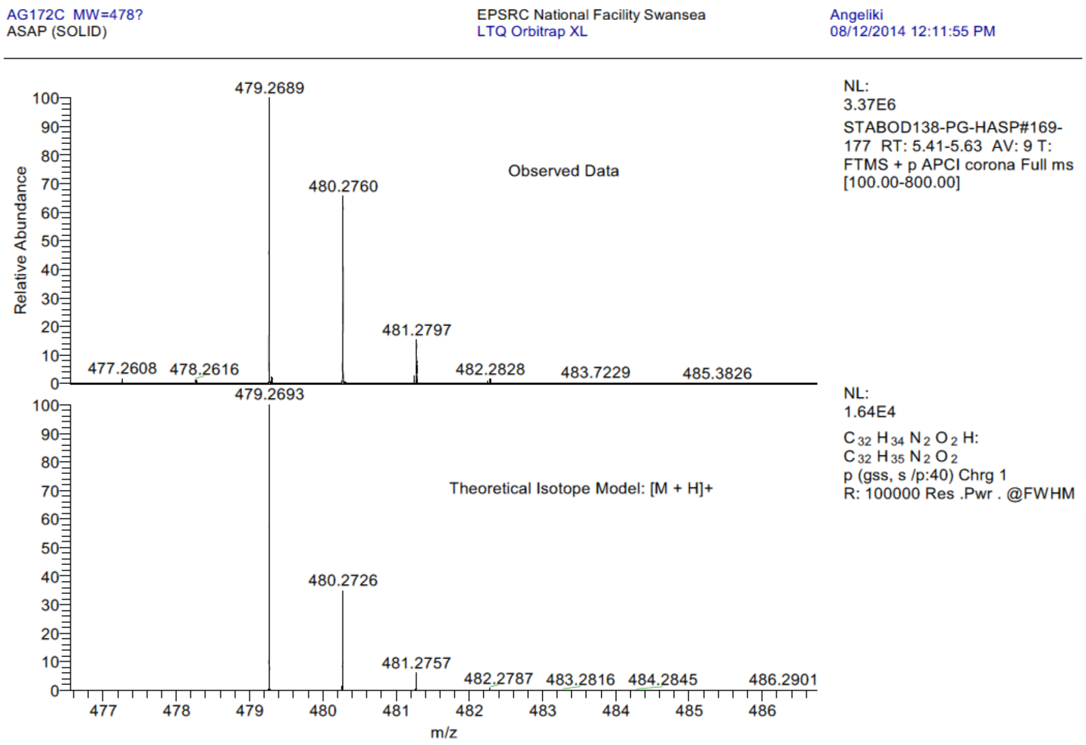

Figure S6 Mass spectrum of **3**.

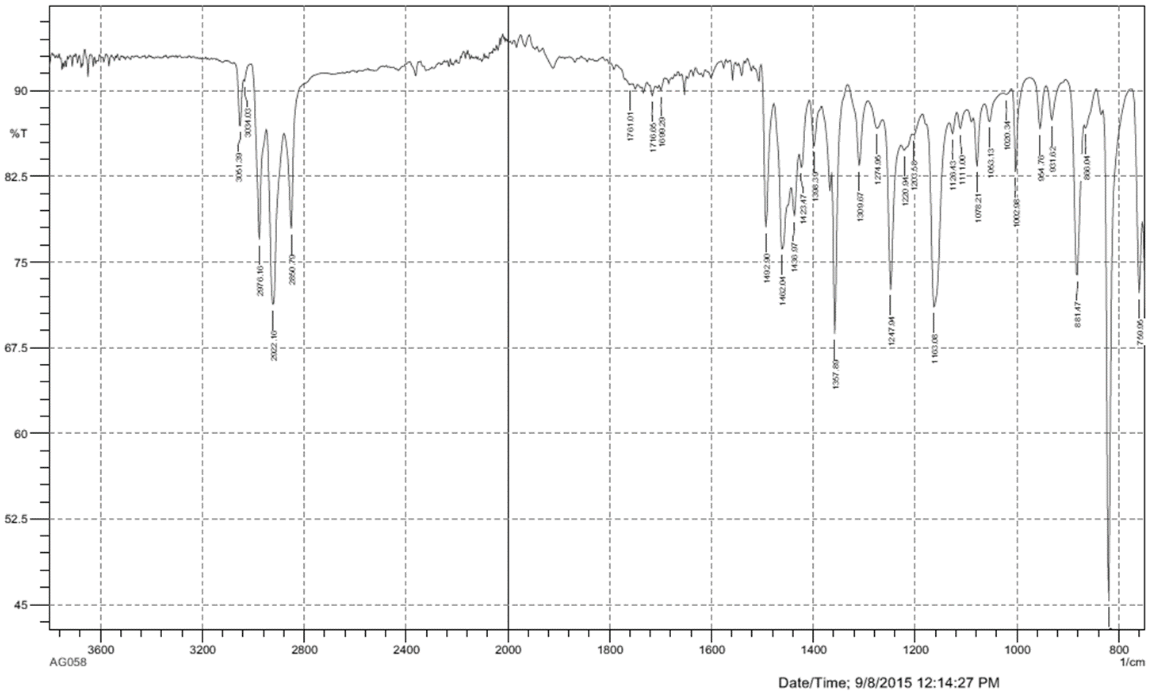

Figure S7 IR spectrum of **3**.

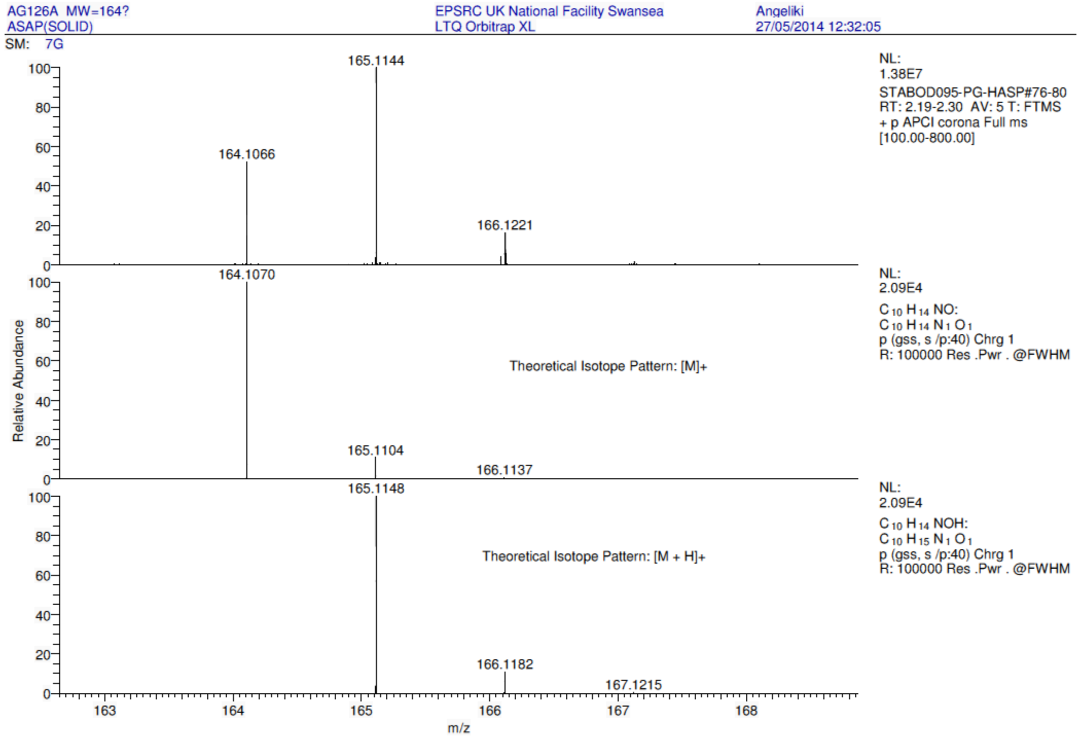

Figure S8 Mass spectrum of **4** from **15**.

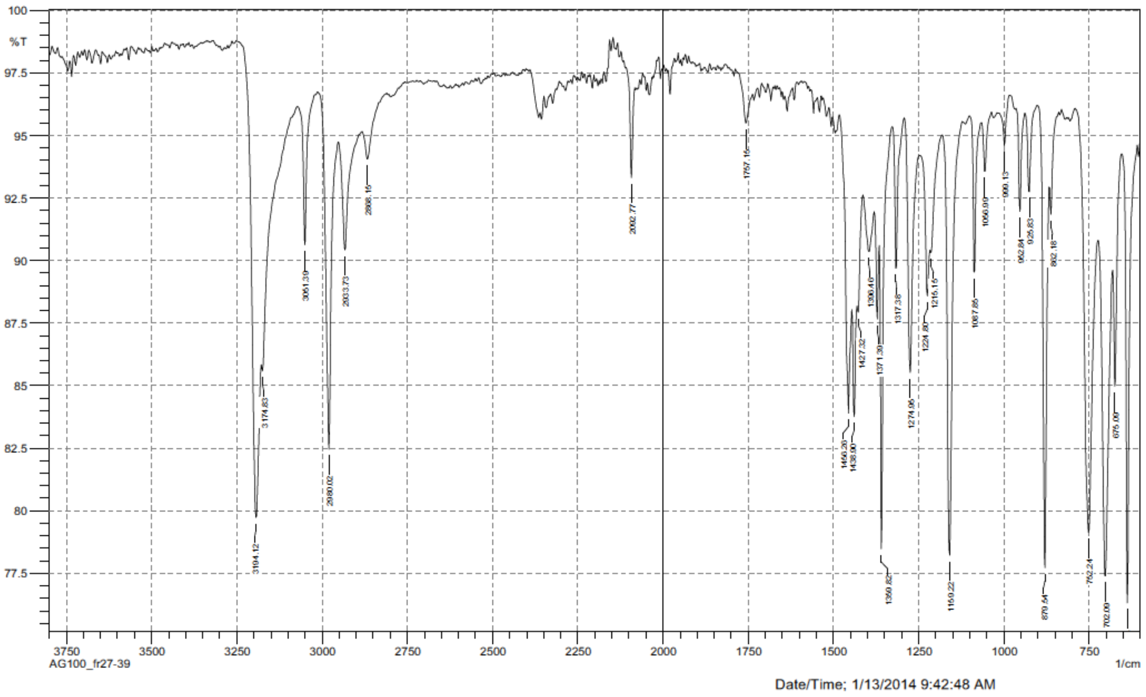

Figure S9 IR spectrum of **4**.

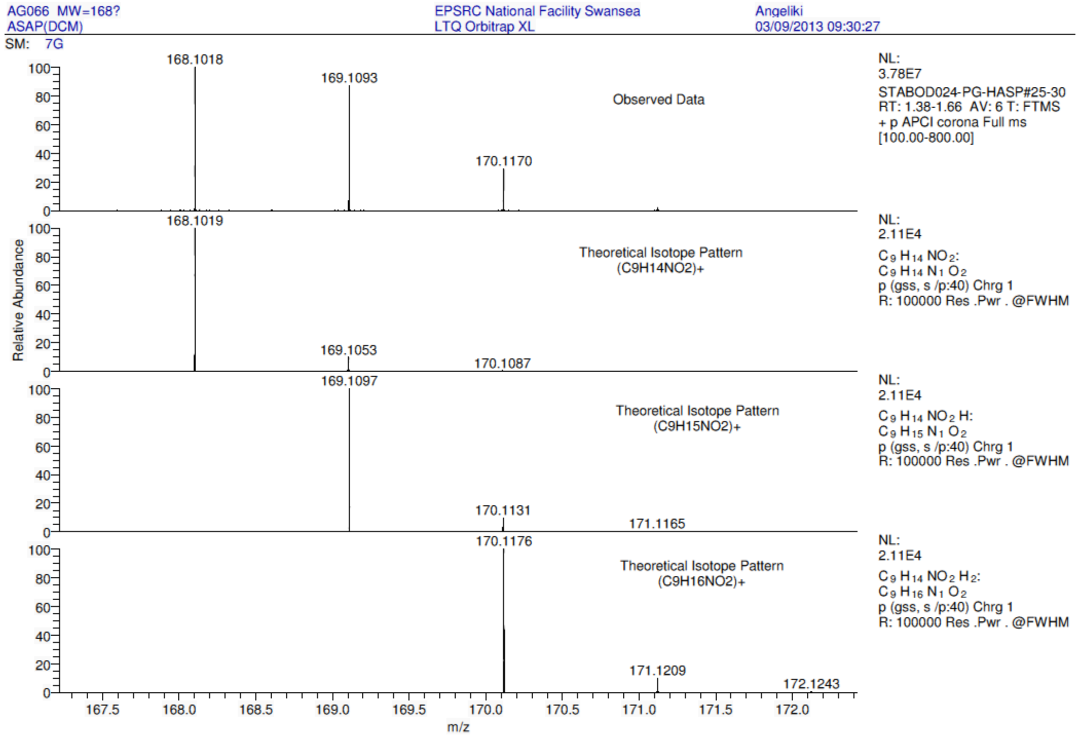

Figure S10 Mass spectrum of **5** from **12**.

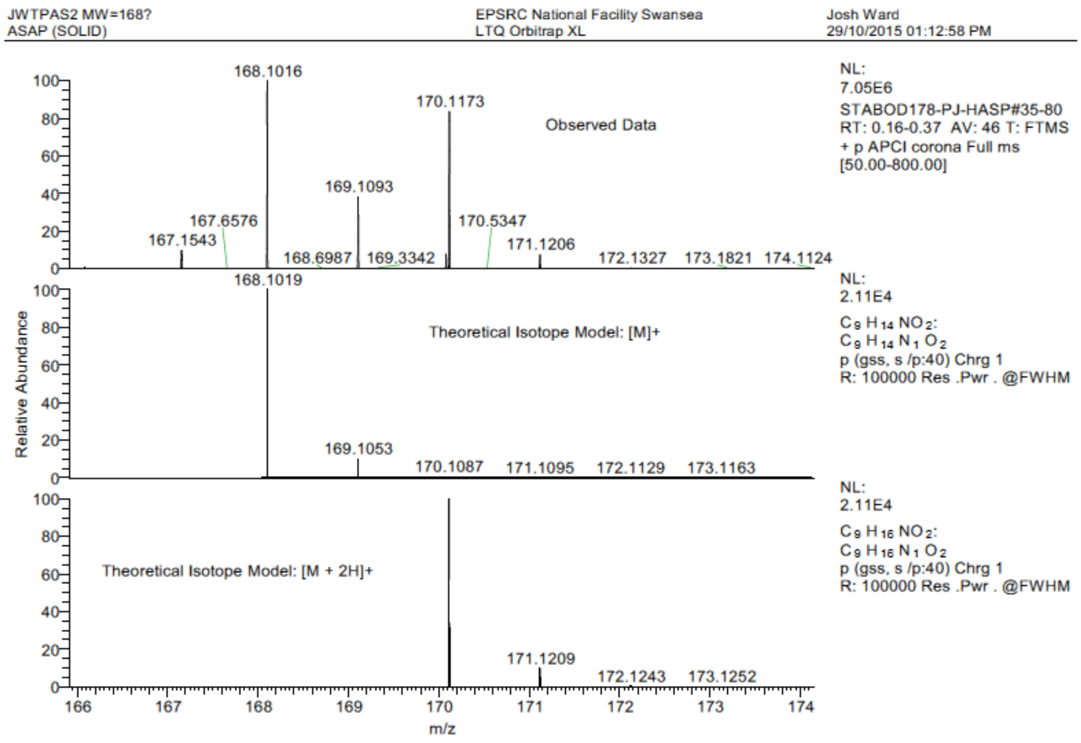

Figure S11 Mass spectrum of **5** from **10**.

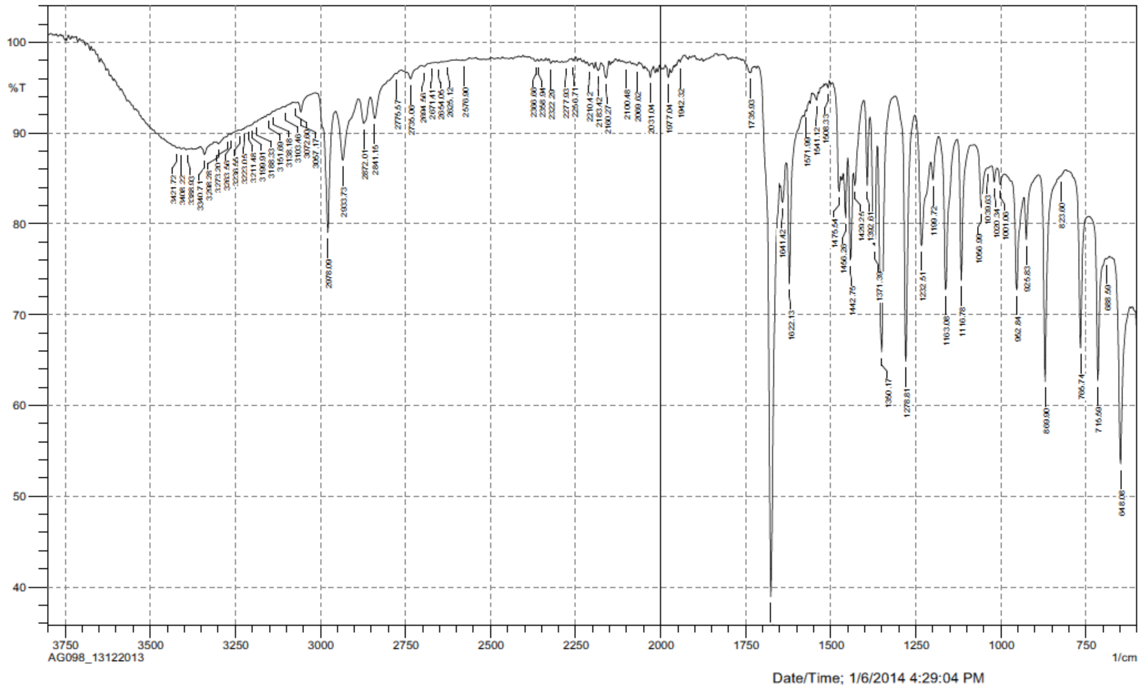

Figure S12 IR spectrum of **5**.

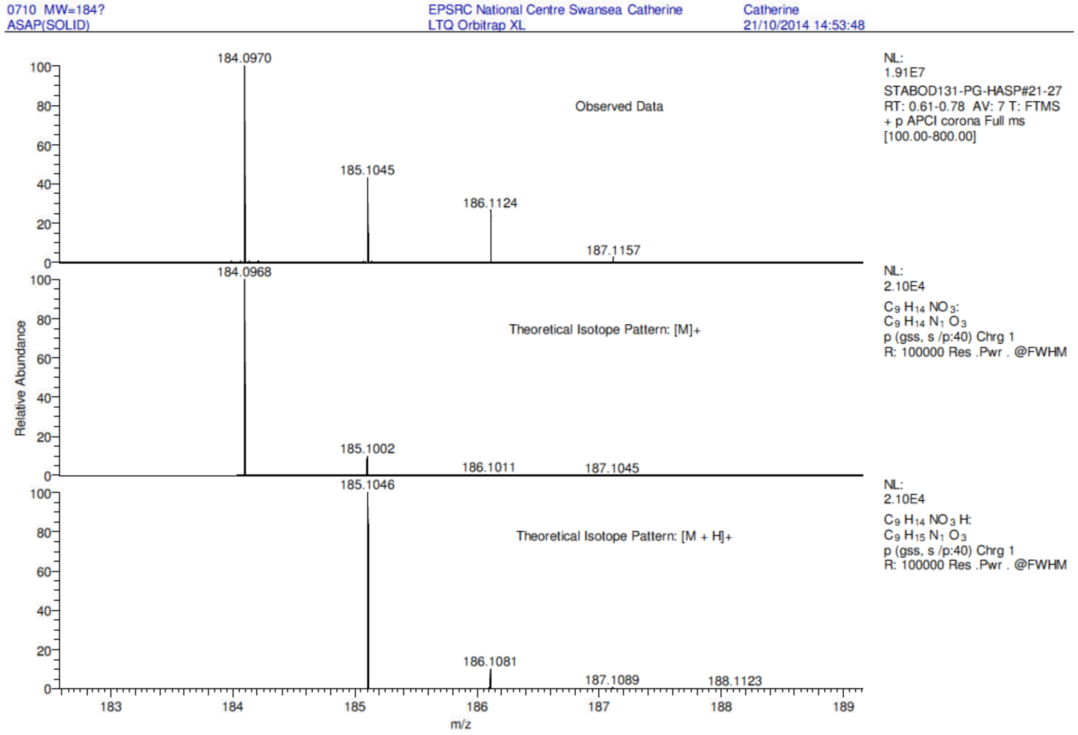

Figure S13 Mass spectrum of **6**.

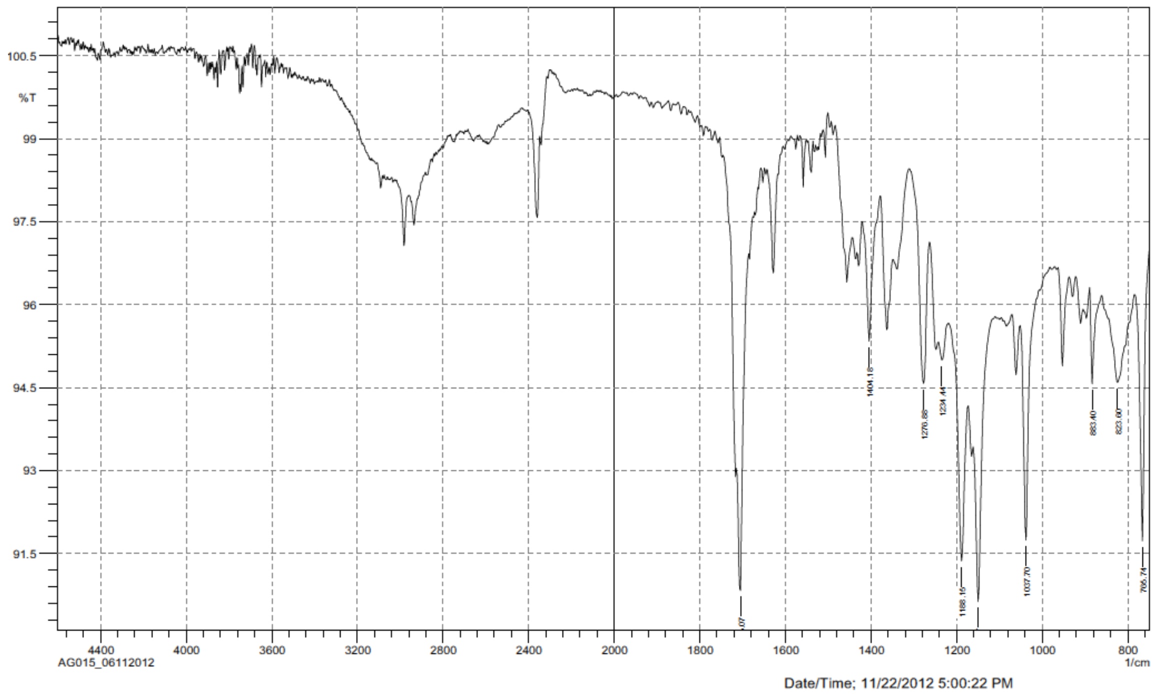

Figure S14 IR spectrum of **6**.

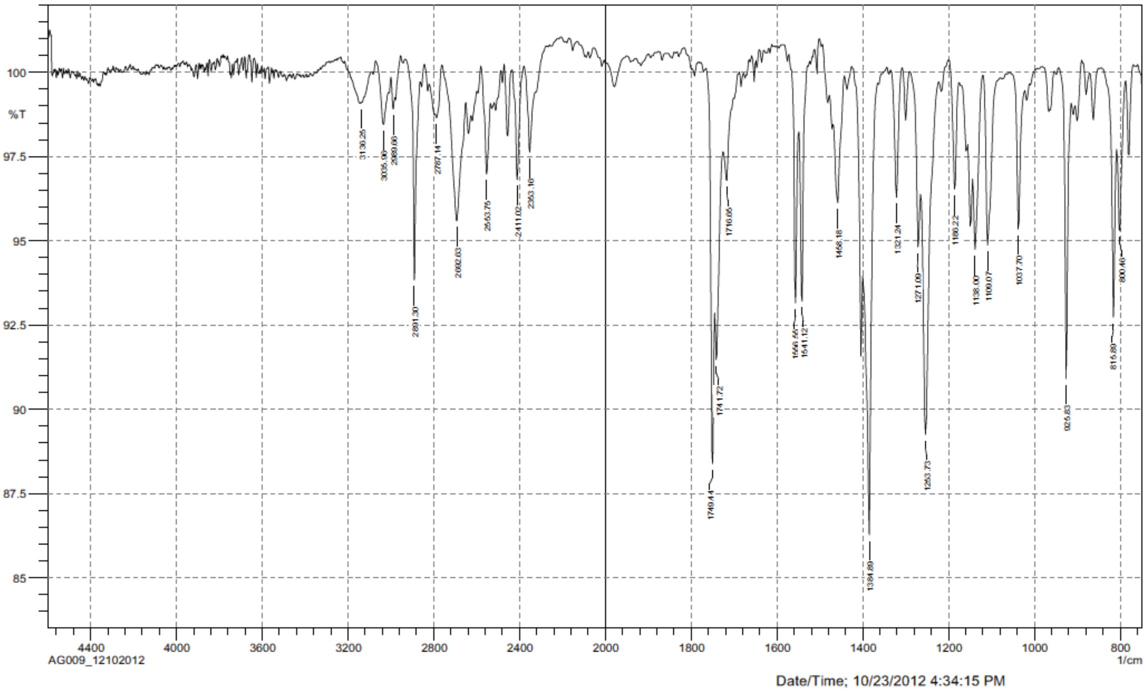

Figure S15 IR spectrum of the first product of reaction from **7** to **8**.

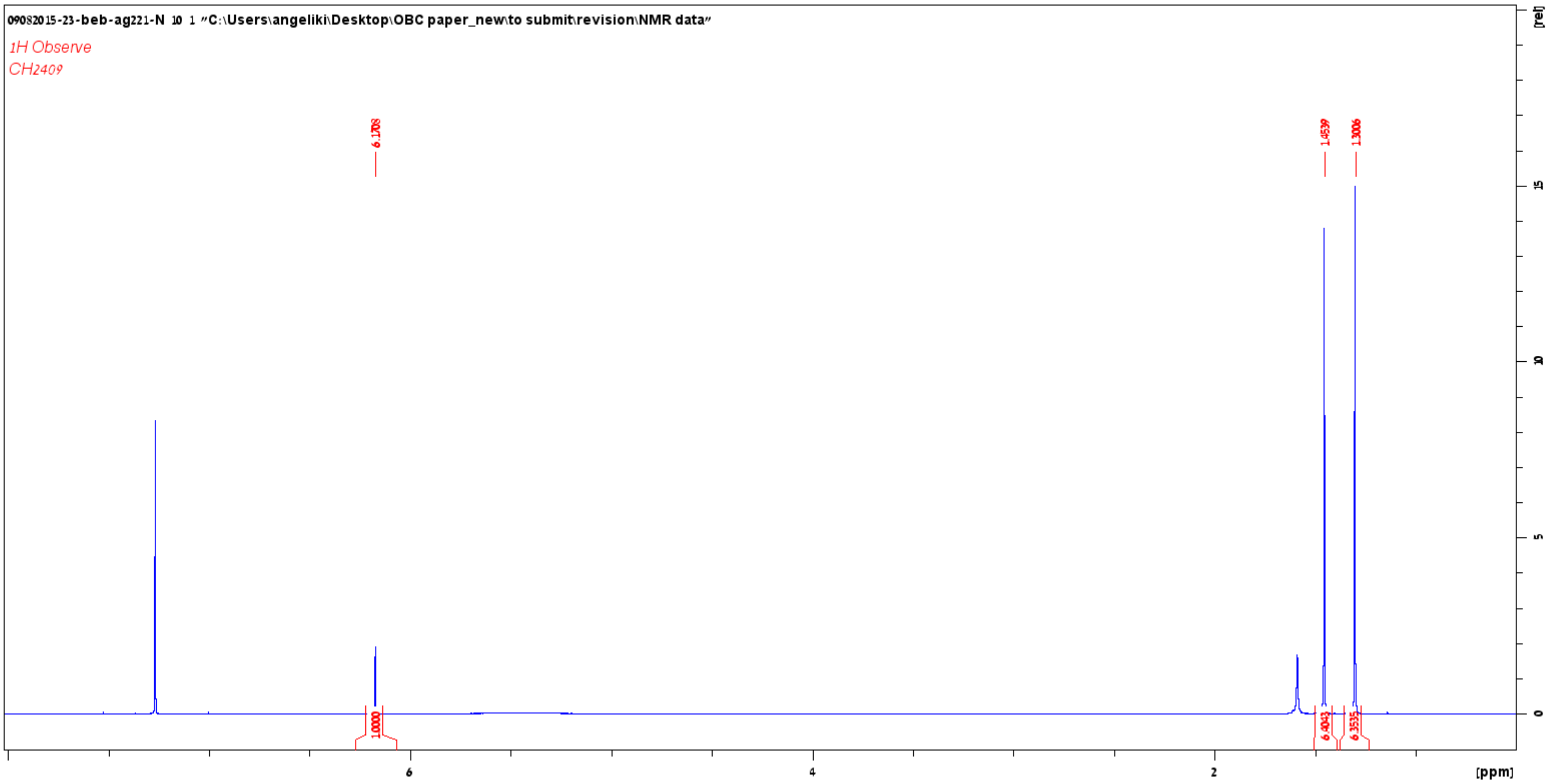

Figure S16 ^{1}H NMR spectrum of **8**.

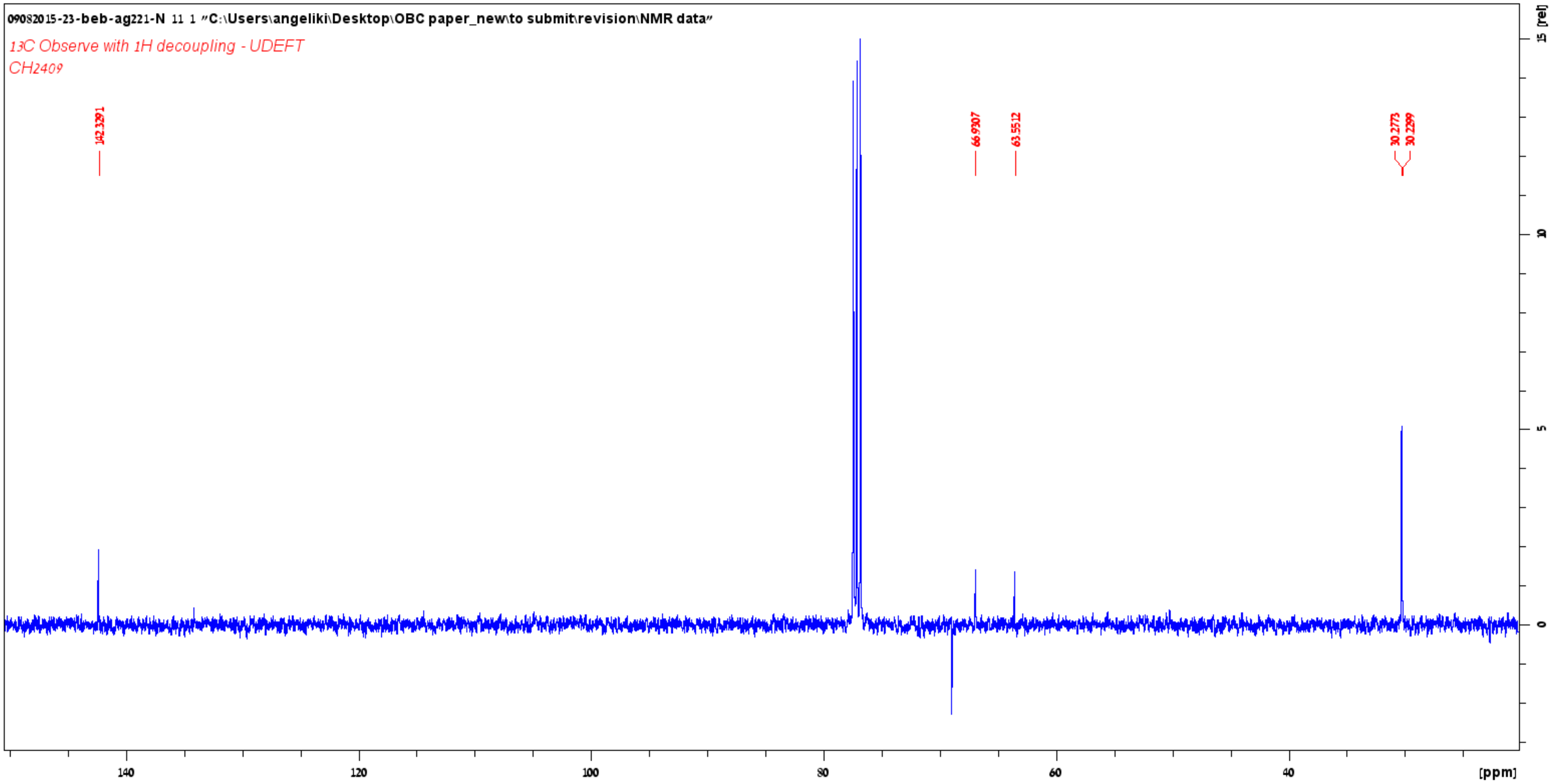

Figure S17 ^{13}C NMR with ^{1}H decoupling spectrum of **8**.

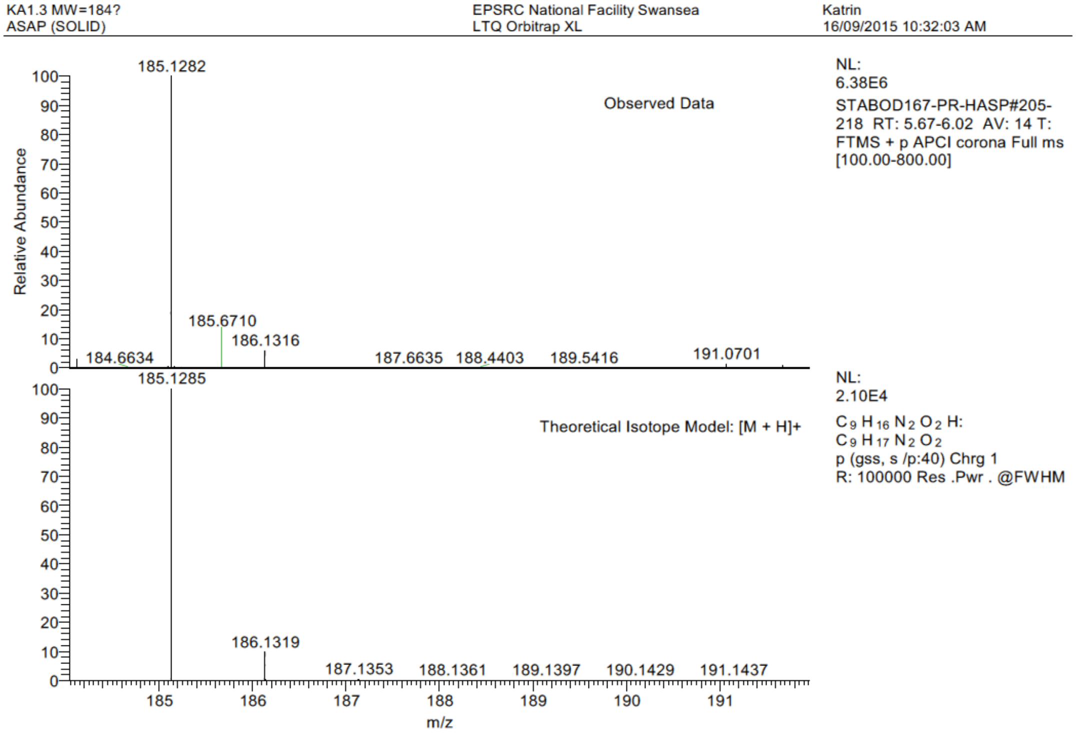

Figure S18 Mass spectrum of **9**.

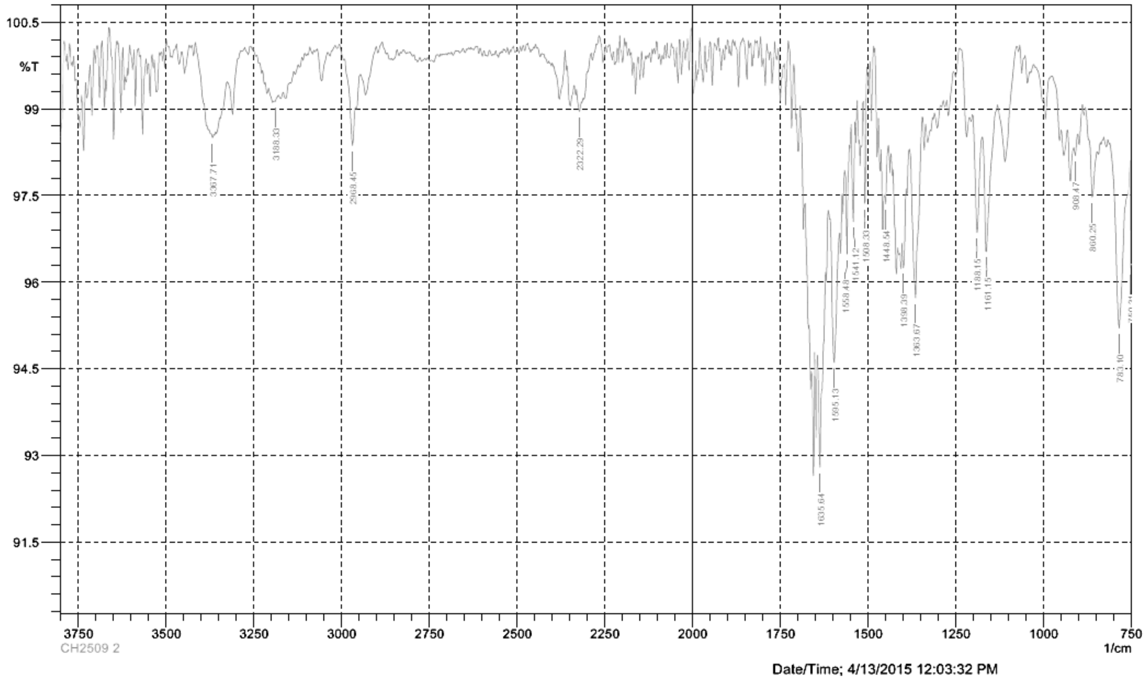

Figure S19 IR spectrum of **9**.

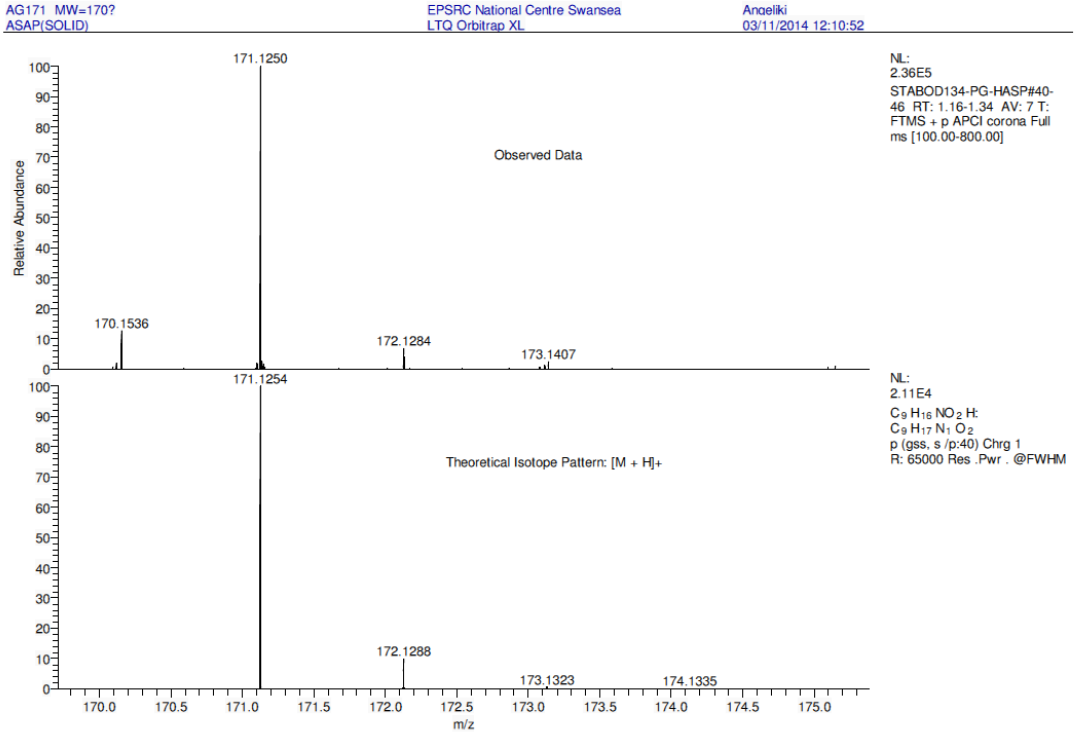

Figure S20 Mass spectrum of **10**.

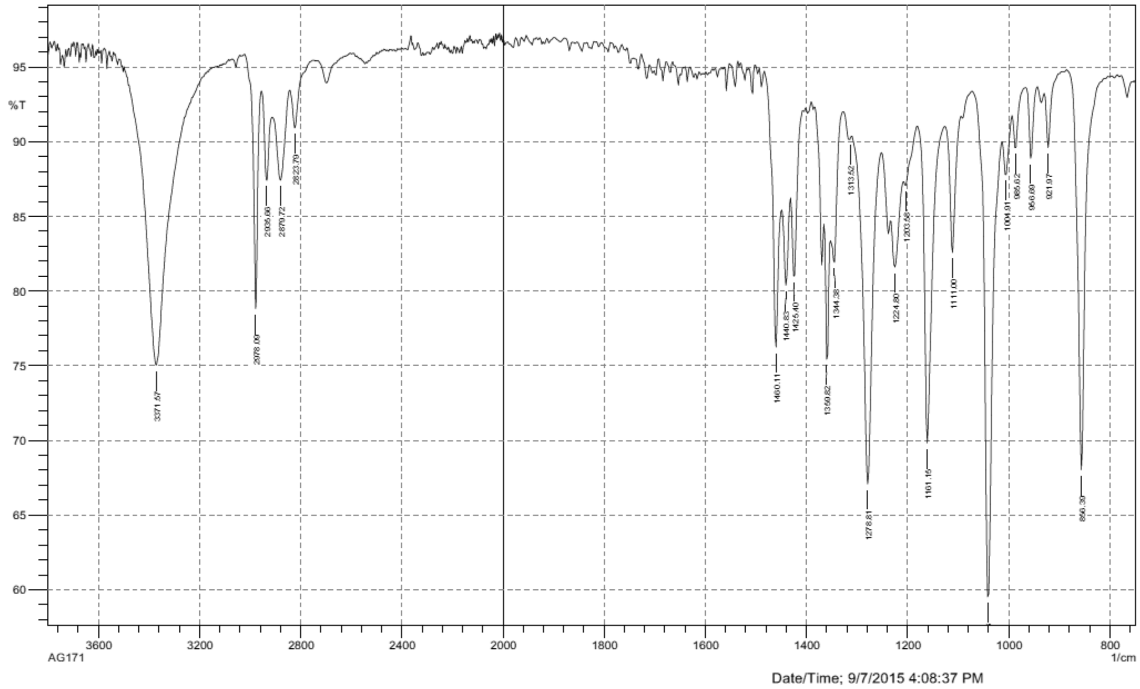

Figure S21 IR spectrum of **10**.

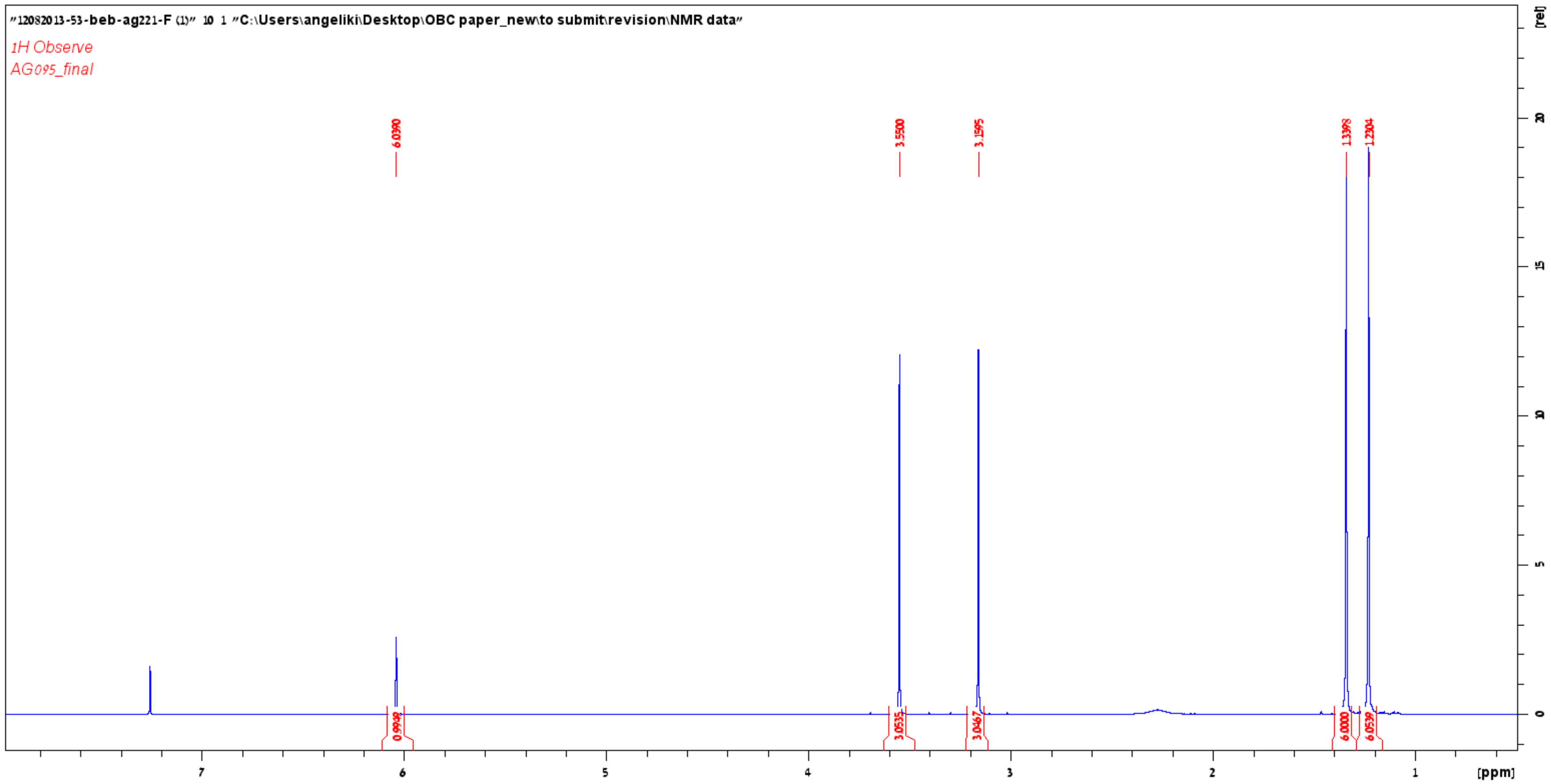

Figure S22 ^{1}H NMR spectrum of **11**.

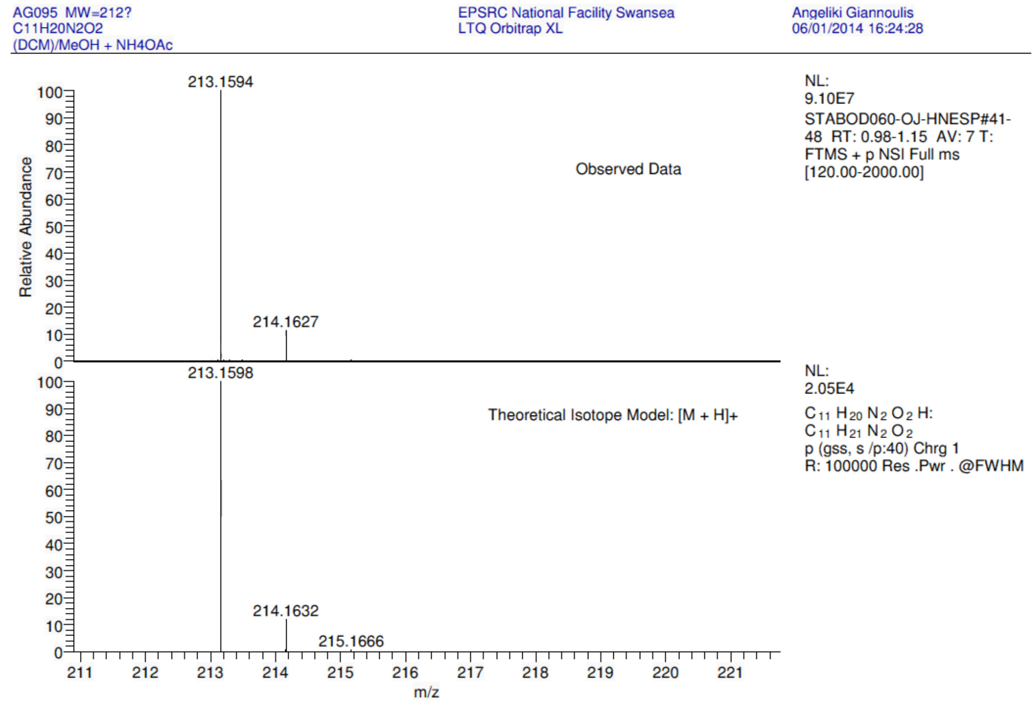

Figure S23 Mass spectrum of **11**.

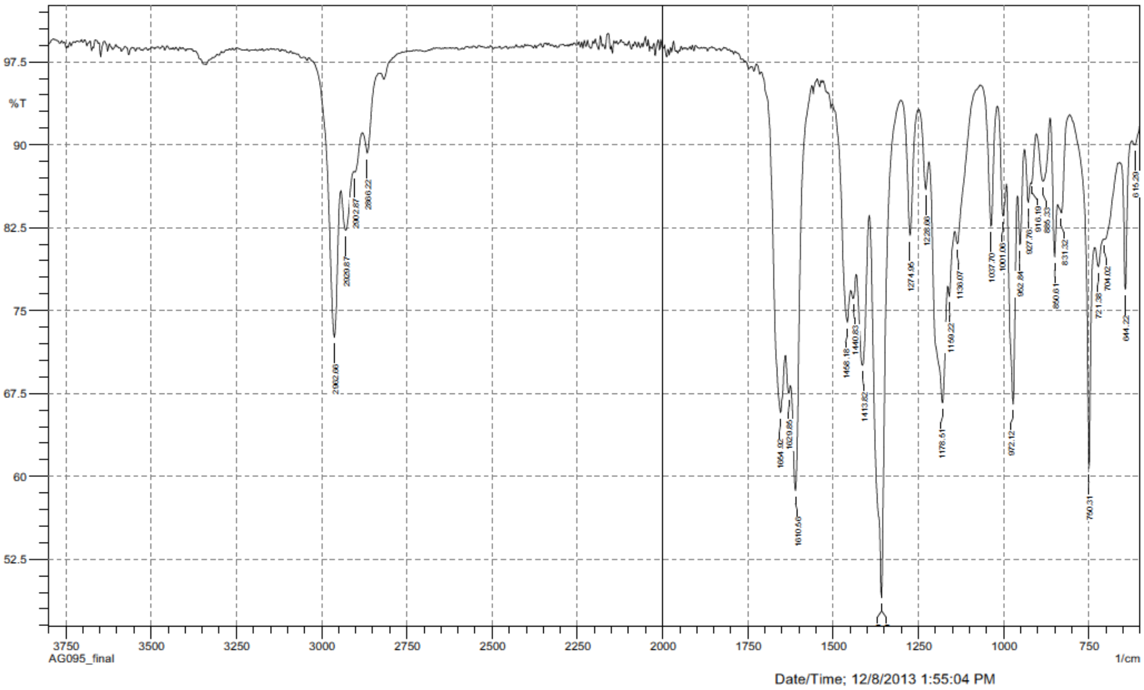

Figure S24 IR spectrum of **11**.

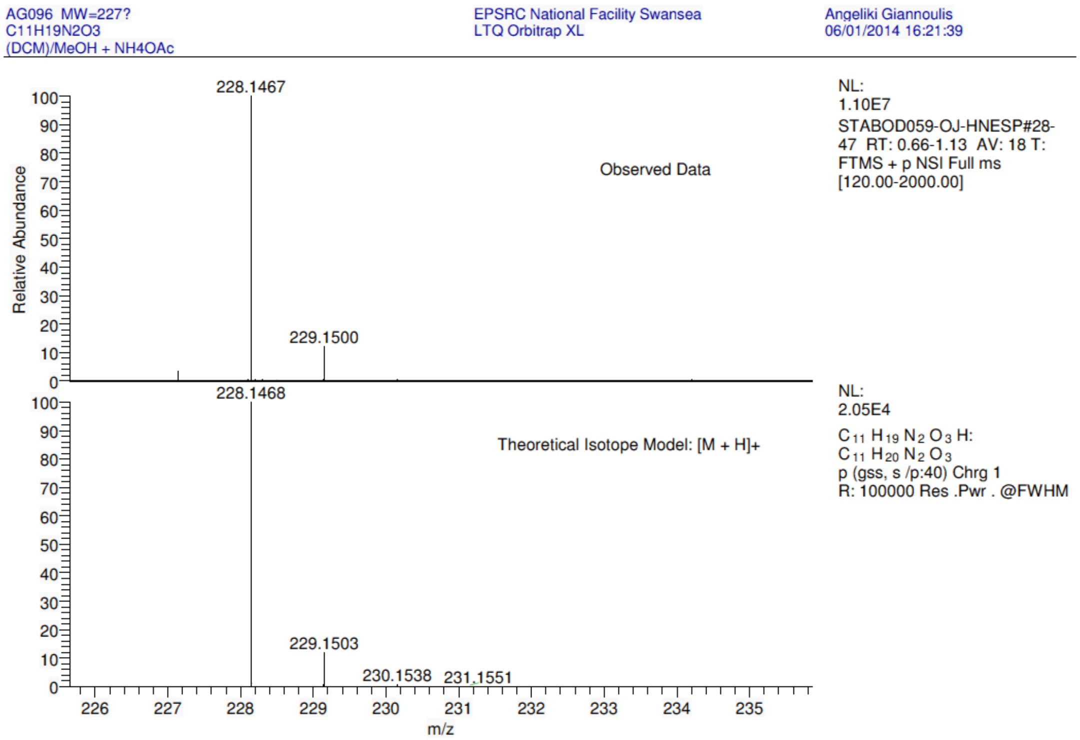

Figure S25 Mass spectrum of **12.**

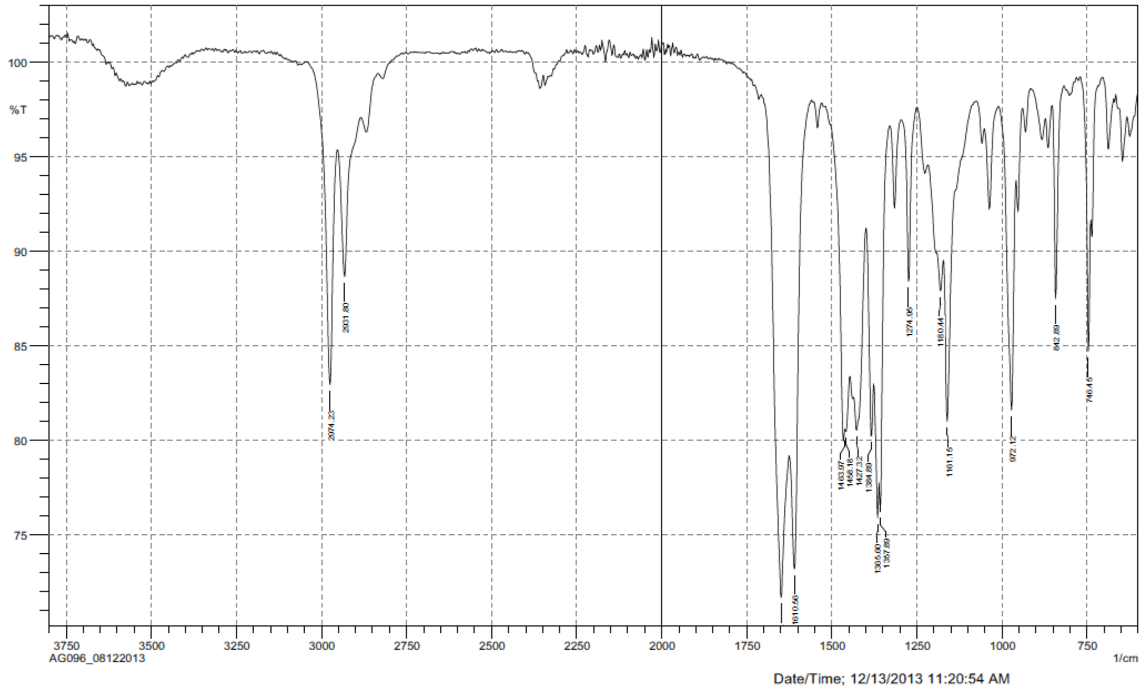

Figure S26 IR spectrum of **12**.

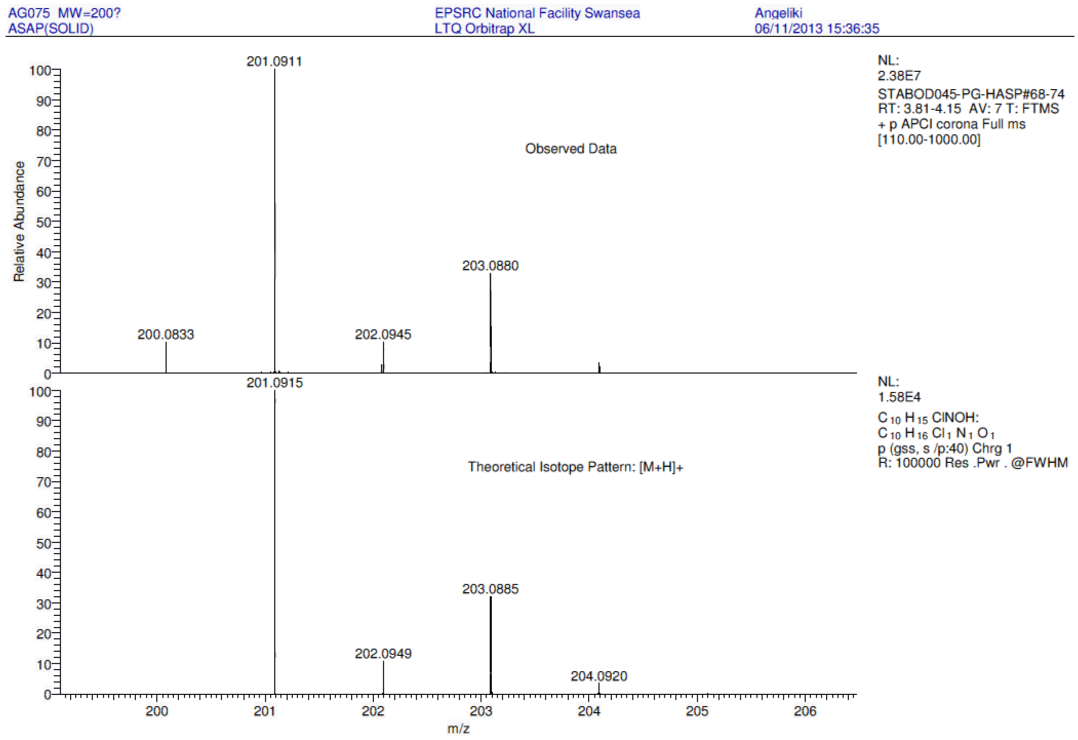

Figure S27 Mass spectrum of **14**.

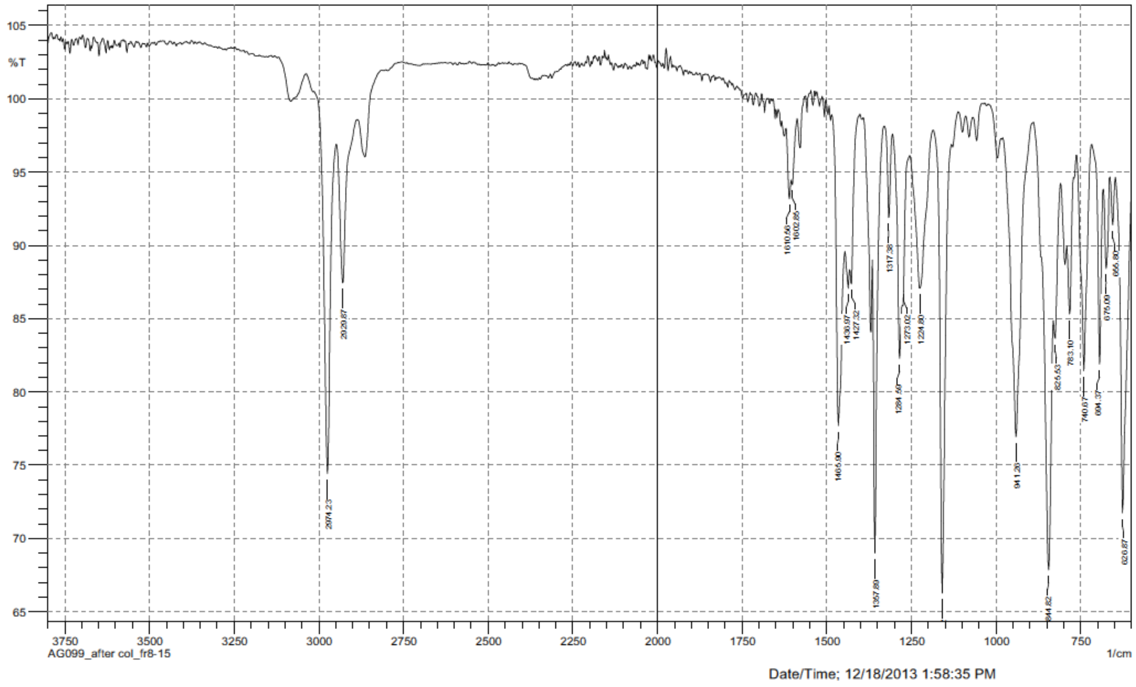

Figure S28 IR spectrum of **14**.

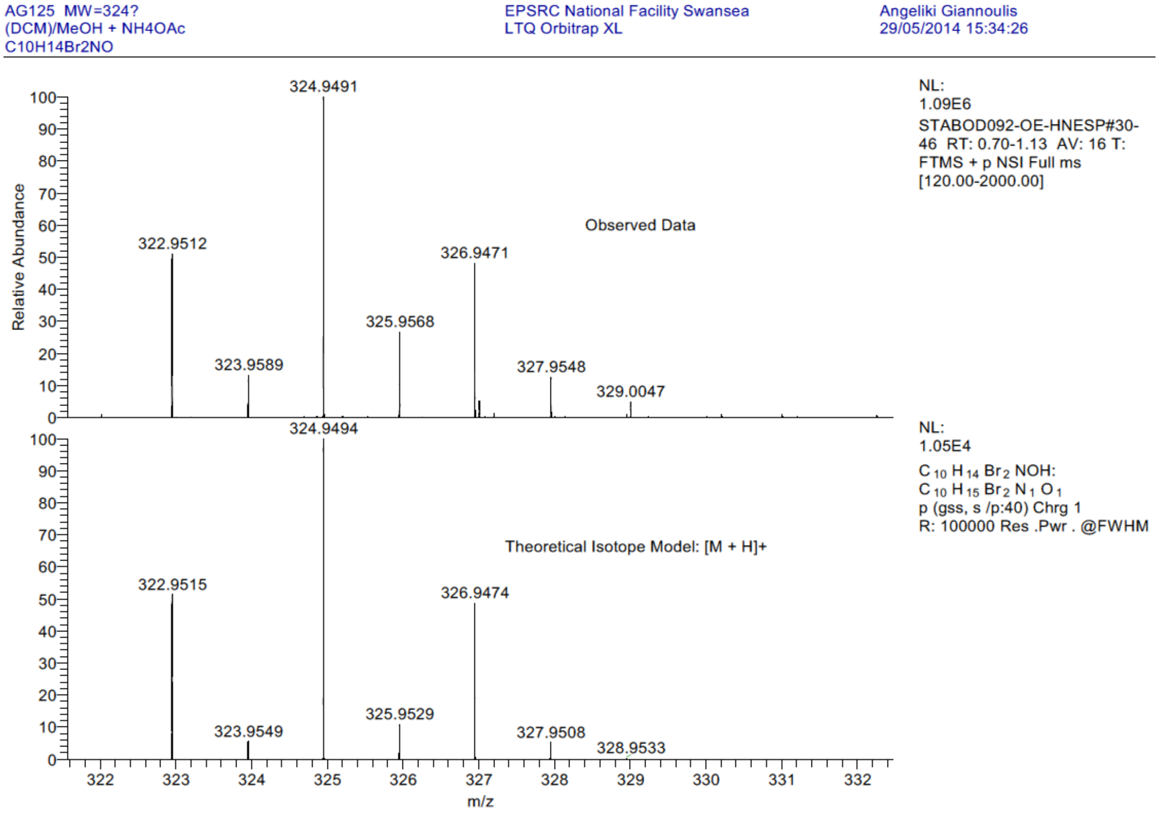

Figure S29 Mass spectrum of **15**.

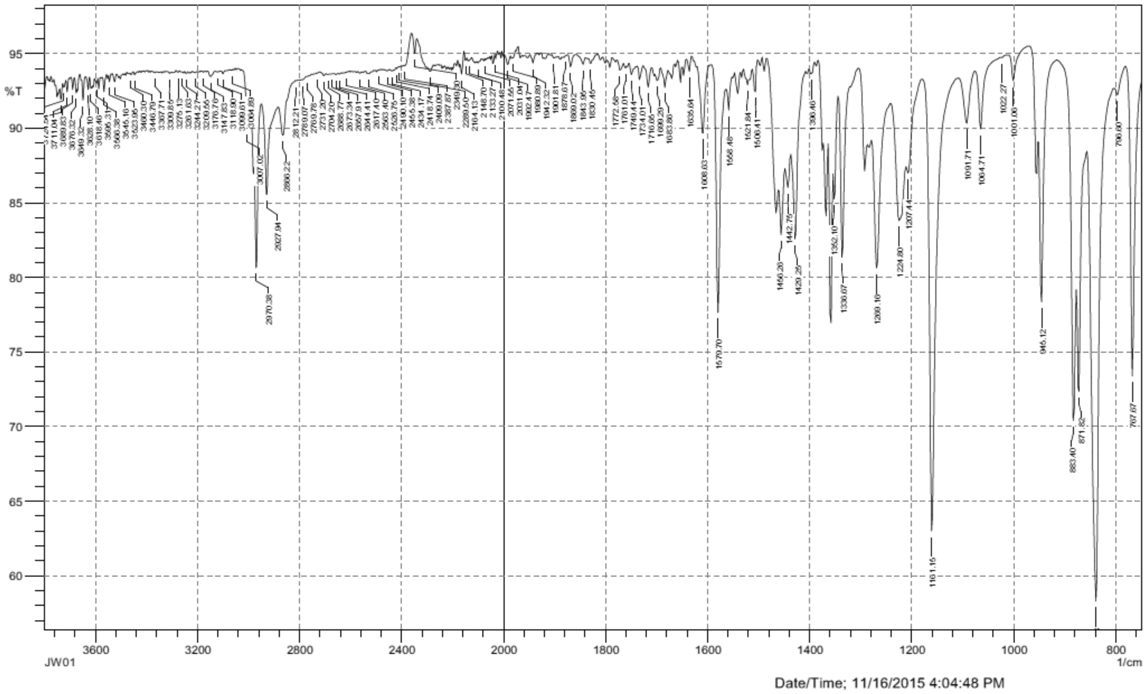

Figure S30 IR spectrum of **15**.

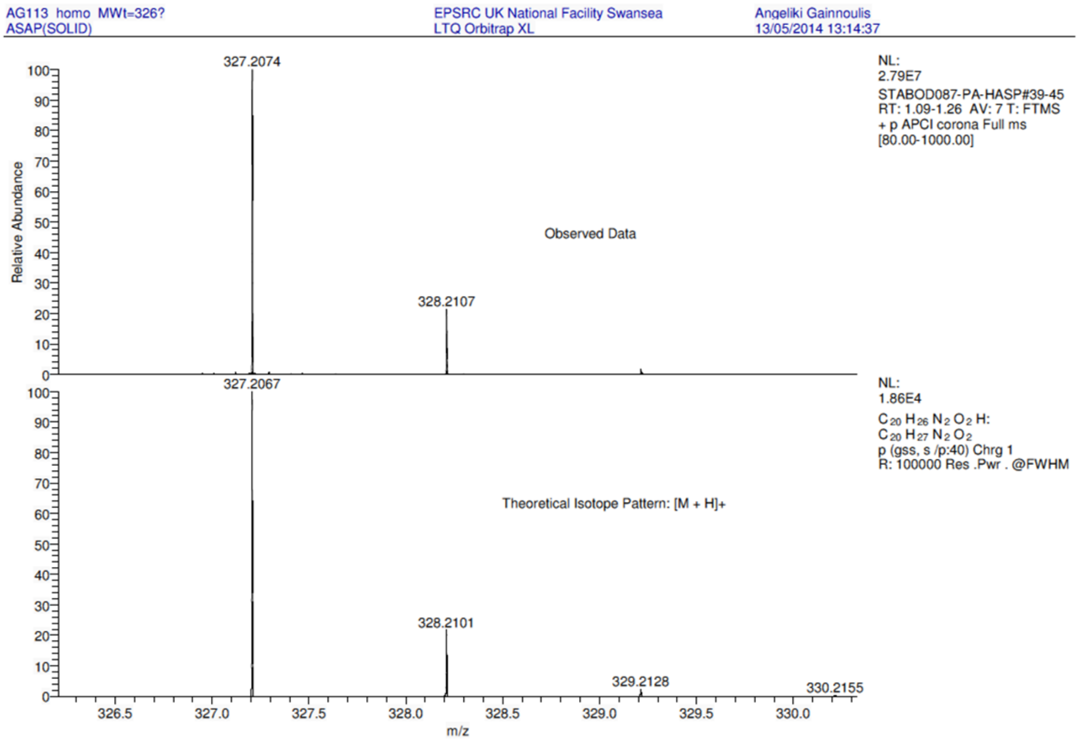

Figure S31 Mass spectrum of **20**.

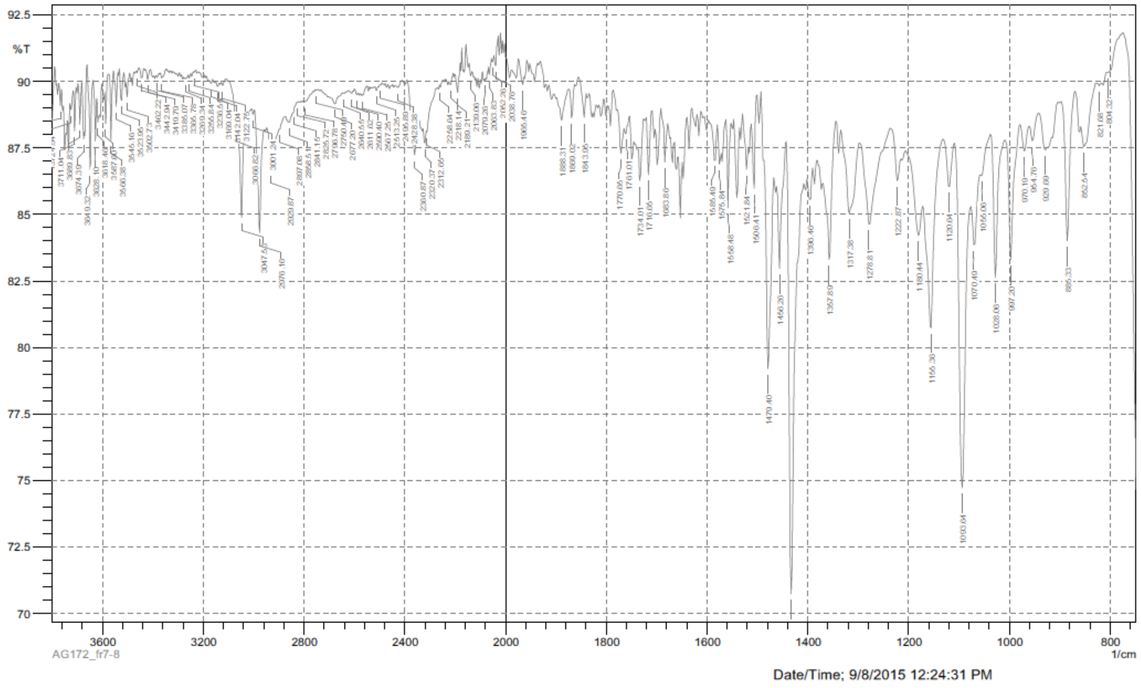

Figure S32 IR spectrum of **20**.

Experiment repeats

Compound **8:** 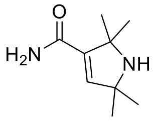

Run	ID	SM* (g/mmol)	NH_3 (mL/mmol)	Yield** (g/%)	Reaction time	Temperature
1	AG008	22.8/58.0	71/1286	7.5/**58**	2 h	rt
2	KA1.2	23.0/58.0	79/1431	5.1/38	2 h	rt

*SM refers to the intermediate compound from **7** to **8**
**The yield is with respect to the starting material (over two steps)

Compound **9**: 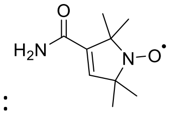

Run	ID	SM (g/mmol)	EDTA* (g/mmol)	$Na_2WO_4 \cdot 2H_2O$ (g/mmol)	H_2O_2 (mL/mmol)	Yield (g/%)	Reaction time	Temperature
1	AG011	7.5/45	0.42/1.4	0.4/1.27	8.3/268	1.8/22	5 min then 6 d	rt then 4 °C
2	KA1.3	5.0/30	0.3/0.9	0.3/0.85	5.5/178	4.0/**73**	5 min then 5 d	rt then 4 °C

*For AG011 was used EDTA, while for KA1.3 $Na_2 \cdot EDTA \cdot 2H_2O$ was used

Compound **6 (TPC)**: 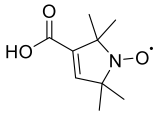

Run	ID	SM (g/mmol)	10% NaOH (mL/mmol)	Yield (g/%)	Reaction time	Temperature
1	AG015	1.78/9.7	45/112	0.7/39	2 h	reflux
2	KA1.4	4.0/22	100/249	2.1/**52**	2 h	reflux
3	JWSTEP3TPC	1.5/8.2	75	0.4/28	2 h	reflux

Compound **10**: 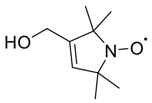

Run	ID	SM (g/mmol)	Red-Al® (mL/mmol)	Yield (g/%)	Reaction time	Temperature
1	AG171	1.0/5.4	6/19.8	0.35/40	1.5 h	55 °C
2	KA2_1	2.0/10.8	12/39	1.6/**87**	1.5 h	55 °C

Compound **11**: 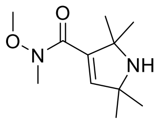

Run	ID	SM* (g/mmol)	Me(MeO)NH.HCl (g/mmol)	Yield** (g/%)	Reaction time	Temperature
1	AG061	10/25.0	3.0/30	3.8/71	overnight +6 h	rt+50 °C
2	AG064	13.2/33.5	3.9/40	3.1/44	48 h+6 h	rt+50 °C
3	AG095	24.7/62.8	7.3/75.4	6.7/50	48 h+6 h	rt+50 °C
4	AG119	37.1/94.0	11.0/113.0	5.6/28	overnight +6 h	rt+50 °C
5	AG122	29.8/75.8	8.8/90.1	4.7/29	5 d	rt
6	AG163	33.5/85.2	9.9/101.6	13.0/**65**	7 d	rt

*SM refers to the intermediate compound from **7** to **8**

**The yield is with respect to the starting material (over two steps)

Compound **12**: 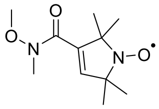

Run	ID	SM (g/mmol)	EDTA* (g/mmol)	$Na_2WO_4 \cdot 2H_2O$ (g/mmol)	H_2O_2 (mL/mmol)	Yield (g/%)	Reaction time	Temperature
1	AG065	3.1/14.8	0.14/0.5	0.14/0.4	2.75/91	2.5/**75**	8 min then 48 h	rt then rt
2	AG096	6.7/31.6	0.33/1.0	0.30/0.9	6.3/211	3.9/55	8 min then 5 d	rt then rt
3	AG120	5.6/26.3	0.28/0.8	0.25/0.75	5.2/210	3.6/60	8 min then 8 d	rt then rt
4	AG123	4.7/22.3	0.24/0.7	0.21/0.64	4.4/147	3.3/65	8 min then 10 d	rt then rt
5	AG164	13.1/62	0.66/2.0	0.59/1.79	12/400	4.8/34	9 min then 8 d	rt then rt

*For AG065 EDTA was used, while for AG096, AG120, AG123 and AG164 $Na_2 \cdot EDTA \cdot 2H_2O$ was used.

Compound **5** from **12**: 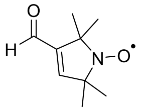

Run	ID	SM (g/mmol)	DIBAL* (mL/mmol)	Yield (g/%)	Reaction time**	Temperature
1	AG066	2.5/11.0	11.3/13.6	1.4/75	50 min	−78 °C
2	AG098	3.9/17.4	18.0/21.6	2.8/**95**	45 min	−78 °C
3	AG124	3.6/15.9	19.6/19.6	1.2/47	35 min	−78 °C
4	AG147	3.3/14.6	18.0/18.0	0.3/12	35 min	−78 °C
5	AG165	4.8/20.9	21.0/25.2	2.4/69	35 min	−78 °C

* For AG066, AG098 and AG165 the concentration of DIBAL was 1.2 M, while for AG124, AG147 1.0 M

** Refers to DIBAL addition time and further stirring

Compound **5** from **10**: 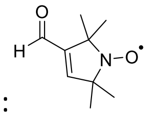

Run	ID	SM (g/mmol)	$C_2O_2Cl_2$ (mL/mmol)	DMSO (mL/mmol)	Yield (g/%)	Reaction time	Temperature
1	JWTPAS2	0.4/2.4	0.25/2.6	0.4/5.6	0.2/**56**	15 min	−65 °C
2	KA2.2	1.6/9.4	0.93/10.3	1.6/22.4	0.7/44	15 min	-60 (to -53) °C

Compound **14**: 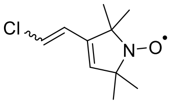

Run	ID	SM (g/mmol)	$ClCH_2P(Cl)(C_6H_5)_3$ (g/mmol)	*n*-BuLi* (mL/mmol)	Yield (g/%)	Reaction time	Temperature
1	AG075	1.4/8.3	11.5/33.0	11.1/29.6	1.6/**98**	30 min	0 °C
2	AG099	1.9/11.4	15.7/45.2	18.2/41.7	0.3/15	45 min	0 °C
3	AG102	1.0/5.7	8.0/23.2	12.4/21	0.1/11	40 min	0 °C
4	AG166	2.4/14.5	5.5/15.9	9.5/16.1	3.0/96	2 h then 1 h	rt then 50 °C
5	KA5.1	0.25/1.5	0.6/1.6	1.0/1.6	0.15/49	2 h then 1 h	rt then 50 °C

* *n*-BuLi in hexanes in 2.5 M concentation for AG075, 2.3 M for AG099, 1.7 M for AG102 and AG166 determined after titration with diphenylacetic acid

Compound **15**: 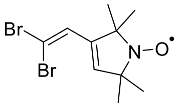

Run	ID	SM (g/mmol)	CBr_4 (g/mmol)	PPh_3 (g/mmol)	Yield (g/%)	Reaction time	Temperature
1	AG125	1.2/7.0	4.6/14.0	7.3/28.0	1.5/**66**	20 min then rt	0 °C then 1 h
2	JW01	0.07/0.4	0.3/0.8	0.4/1.7	0.03/6	15 min	0 °C
3	KA7.1b	0.25/1.5	1.0/3.0	1.6/6.0	0.06/12	15 min	0 °C

Compound **4 (TPA)** from **14**: 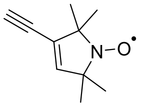

Run	ID	SM (g/mmol)	KO^tBu (g/mmol)	Yield (g/%)	Reaction time	Temperature
1	AG100	0.3/1.6	0.5/4.1	0.06/22	55 °C	2 h
2	AG167	1.0/5.0	1.2/10.9	0.04/5	55 °C	2 h
3	AG169	0.3/1.6	0.5/4.1	0.03/11	55 °C	2 h
4	KA6.1	0.15/0.7	0.5/4.1	0.03/**26**	55 °C	2 h

Compound **4 (TPA)** from **15**: 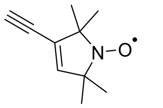

Run	ID	SM (g/mmol)	CBr_4 (g/mmol)	Yield (g/%)	Reaction time	Temperature
1	AG126	0.47/1.5	1.5/2.9	0.02/**10**	45 min then 3 h	-78 °C then rt
